# Non-thermal plasma-assisted rapid hydrogenolysis of polystyrene to high yield ethylene

**DOI:** 10.1038/s41467-022-28563-7

**Published:** 2022-02-16

**Authors:** Libo Yao, Jaelynne King, Dezhen Wu, Jiayang Ma, Jialu Li, Rongxuan Xie, Steven S. C. Chuang, Toshikazu Miyoshi, Zhenmeng Peng

**Affiliations:** 1 Department of Chemical, Biomolecular and Corrosion Engineering, The University of Akron, 200 E Buchtel Avenue, Akron, OH 44325 USA; 2 School of Polymer Science and Polymer Engineering, The University of Akron, 170 University Avenue, Akron, OH 44325 USA

**Keywords:** Pollution remediation, Sustainability

## Abstract

The evergrowing plastic production and the caused concerns of plastic waste accumulation have stimulated the need for waste plastic chemical recycling/valorization. Current methods suffer from harsh reaction conditions and long reaction time. Herein we demonstrate a non-thermal plasma-assisted method for rapid hydrogenolysis of polystyrene (PS) at ambient temperature and atmospheric pressure, generating high yield (>40 wt%) of C_1_–C_3_ hydrocarbons and ethylene being the dominant gas product (Selectivity of ethylene, *S*_C2H4_ > 70%) within ~10 min. The fast reaction kinetics is attributed to highly active hydrogen plasma, which can effectively break bonds in polymer and initiate hydrogenolysis under mild condition. Efficient hydrogenolysis of post-consumer PS materials using this method is also demonstrated, suggesting a promising approach for fast retrieval of small molecular hydrocarbon modules from plastic materials as well as a good capability to process waste plastics in complicated conditions.

## Abstract (web summary)

Current recycling methods suffer from harsh reaction conditions and long reaction times. Here the authors show a non-thermal plasma-assisted method for rapid hydrogenolysis of polystyrene at ambient temperature and atmospheric pressure, generating high yield (>40 wt%) of C1–C3 hydrocarbons.

## Introduction

For over a half century the fast development of plastic industry has been contributing greatly to the prosperity of human society. The global production of plastics has reached 400 million tons in 2018 and is still quickly growing, with the annual production being projected to double within two decades^1^. Accompanied by the big demand for plastic products, more and more plastic wastes (PWs) have been generated. It is reported that more than 300 million tons of PWs were generated in 2015 alone^2^. As of date only less than 20% of these PWs can be recycled, with the rest ending up in landfills and incineration or being directly released to environment^3^

Mechanical process, which incorporates shredding, heating, and remolding of plastics, remained as the primary recycle method^3^. Despite the attraction for “closed-loop cycles”, this method mainly produces “downgraded” plastics which have inferior properties^4,5^. As a result, the number of reprocessing cycles is limited. Chemical processes including pyrolysis and gasification have been actively investigated for converting PWs to relatively low value-added synthesis gas and carbonaceous materials^6–8^. However, a high operating temperature (700–1300 K) is typically required to overcome the unfavorable thermodynamics and kinetics that makes these methods cost inhibitive. More recently, there have been a few studies on plastics hydrogenolysis. However, harsh reaction conditions of high temperature (600–700 K) and pressure (10–30 bar) and relatively long reaction time (>6 h) were still needed, suggesting low hydrogenolysis kinetics^9–11^.

Polystyrene (PS) has been widely used as food packages and foams and is among the top five most-produced plastic materials, other than polyethylene (PE), polypropylene (PP), polyethylene terephthalate (PET), and polyvinyl chloride (PVC), accounting for about 10% of global production^12^. Despite some active research efforts^13–15^, few successes have been achieved in recycling this plastic material. As a matter of fact, only 1% of PS waste was recovered in US in 2015^12^. This comes from complex situations for separation (blended products and abundant sources of contaminants) and sorting (diversified density range 0.02–1.1 g/cm^3^) of PS wastes that restrain their collection and the inability to process wastes under aforementioned complicated conditions. To overcome these practical challenges and claim better energy advantage, a method with the versatility to effectively depolymerize PS material under mild reaction conditions is needed.

Herein, we report a non-thermal plasma-enabled method which allows efficient hydrogenolysis of pure and post-consumer PS under ambient temperature and atmospheric pressure condition. Non-thermal H_2_ plasma provides a unique medium for generating reactive hydrogen species, primarily in form of ions and radicals, which can effectively break the C–C bond in the polymeric structures and drive kinetically unfavorable reactions under mild reaction conditions^16,17^. In plastic pyrolysis, DBD plasma was also implemented to alter product selectivity^18–20^, but the process is still subject to high temperature (>773 K) together with extra power input which makes it less energetically attractive. This method achieves fast depolymerization of PS and generates C_1_–C_3_ hydrocarbons as major gas products. Interestingly, ethylene, an important precursor in the polymer industry, can be produced at a yield as high as 38 wt%. Moreover, this non-thermal plasma-assisted hydrogenolysis method allows direct valorization of post-consumer PS products without a need for pretreatments and shows minimal influences by the contaminants and impurities. These findings reveal a great promise of this method for PWs valorization application.

## Results

### Time-dependent plasma-assisted PS hydrogenolysis

**Fig. 1:**
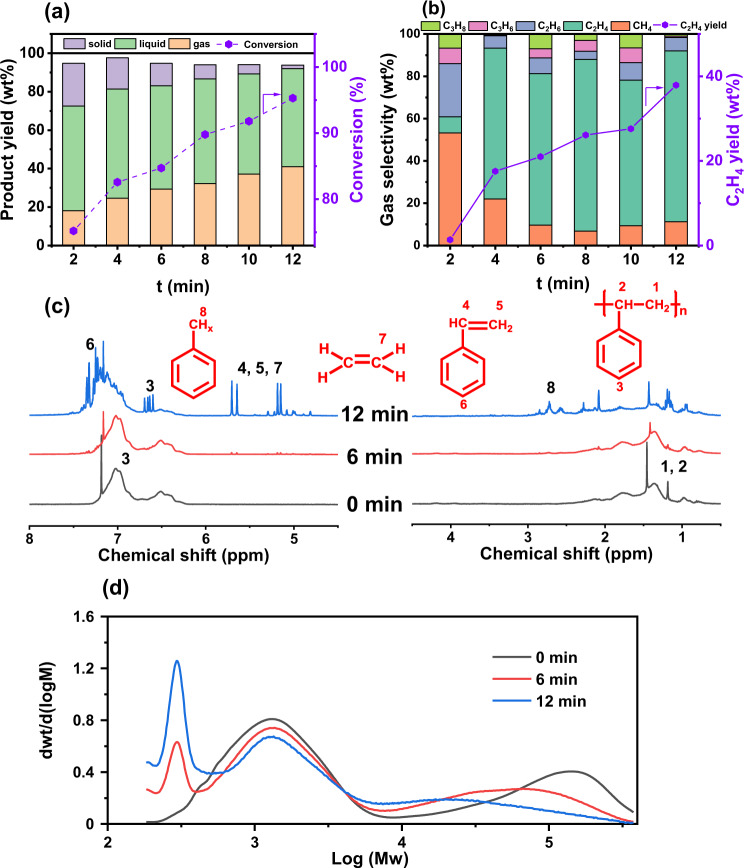
Time-dependent reaction properties of plasma-assisted PS hydrogenolysis. **a** Product yield (*Y*) and conversion (*X*) and (**b**) gas product selectivity and ethylene yield as a function of reaction time (*t*). **c** ^1^H NMR spectra and (**d**) GPC spectra of liquid products after different reaction time (PS-0min, PS-6min, and PS-12min). Reaction conditions: ν_H2_ = 100 ml/min, *P*_H2_ = 101 kPa, *P* = 90 W.

The non-thermal plasma-assisted PS hydrogenolysis experiments were conducted in a continuous flow fixed-bed reactor equipped with a dielectric barrier discharge (DBD) plasma generator (“Methods”, Supplementary Fig. 1). The reaction results in the generation of gas, liquid, and solid products (Fig. 1a), which adds up to over 93 wt% of the total mass recovered (Supplementary Note 1 and Supplementary Tables 1, 2), with a few percent of mass loss during the collection of liquid and solid products. Time-dependent product distribution (denoted as PS-reaction time) shows a continuous increase in the gas products yield (*Y*_g_) with reaction time, which reaches as high as 41.0 wt% after 12 min of reaction. Meanwhile, the solid phase keeps deminishing until to a minimal amount and the liquid products are obtained at a yield (*Y*_l_) ranging from 50 wt% to 56 wt% throughout the experiment. More than 95% of PS is converted to gas and liquid products within 12 min of reaction (Fig. 1a), indicating a fast hydrogenolysis rate. Online GC-MS analyses of the gas effluents find C_1_–C_3_ hydrocarbons as the major gas products (Fig. 1b). Except at the beginning of the reaction when methane is the dominant gas product with 53.2 wt% selectivity at *t* = 2 min, ethylene is found as the primary gas product after a longer reaction time. The ethylene selectivity (*S*_C2H4_) can reach as high as >70%, equivalent to 38 wt% yield among all products (PS-12min). This result confirms the efficient conversion of PS to gaseous hydrocarbons, especially the more valuable ethylene, via plasma-assisted hydrogenolysis.

The liquid products were collected using CHCl_3_ extractant from the resultant liquid–solid mixture after reaction. ^1^H NMR spectra of the obtained liquid products indicate the transformation of polystyrene to styrene-based monomers and oligomers which is evidenced by the emergence of sharp, dense peaks in response to aromatic rings (8–6 ppm), vinyl groups (6–5 ppm) and H_α_ with respect to aromatic functional groups (3–2 ppm), suggesting the significant extent of depolymerization in the liquid components (Fig. 1c)^21^. The monomer and oligomer formation is further confirmed by ^13^C NMR spectroscopy (Supplementary Fig. 4)^22^. GPC was employed to characterize Mw of the liquid product components (Fig. 1d). The Mw profiles of all examined samples exhibit a multi-modal distribution (1–3 modes) owing to the feature of PS feedstock^23^. Two Mw modes, with one at 119,000 Da and the other at 1700 Da, were observed for pure PS (Table 1), amounting to ~35,000 Da average molecular weight. From PS-0min to PS-12min, the hydrogenolysis reaction reduces the high Mw mode by a factor of 10 and the weight percentage of the lower Mw mode also exhibits a significant decrease. Meanwhile, a mode with a much lower Mw of about 300 Da emerges intensively, indicating fast depolymerization of PS within a short reaction time. Characterizations of the solid residues using FT-IR and high-resolution solid-state ^13^C NMR suggest the remaining solids are primarily PS with no detection of other species (Supplementary Figs. 5, 6, Supplementary Table 3).

**Table 1:** A summary of product information after the reaction for different time. Reaction conditions: *t* = 4 min, ν_H2_ = 100 ml/min, *P*_H2_ = 101 kPa, *P* = 90 W.

Entry	*t* (min)	Conversion (%)	Mw (×10^3^ Da)		Yield (wt%)		
					Gas	Liquid^[a]^	Solid
1	0	–	123	1.8	–	–	–
2	2	75.21	119	1.7	18.07	54.52	22.17
3	4	82.59	72	1.9	24.60	56.82	16.23
4	6	84.72	69	1.8	29.32	53.75	11.64
5	8	89.80	57	1.7	32.16	54.45	7.37
6	10	91.80	37	1.8	37.13	52.11	4.81
7	12	95.30	31	1.8	40.99	50.98	1.86

**Fig. 2:**
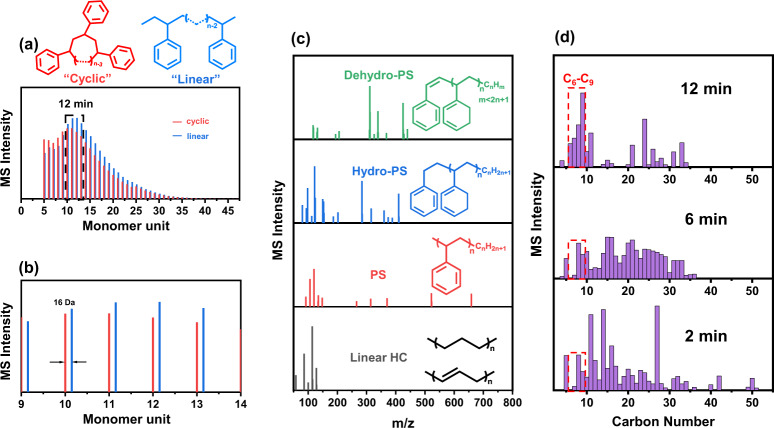
MS characterization of PS hydrogenolysis liquid products. **a** MALDI-MS spectrum of PS-12min with Mw ranging from 500 to 5000 Da. Cyclic and linear polystyrenes were represented in red and blue, respectively. **b** Zoom-in of MALDI spectrum from (**a**), between 9 and 15 monomer units. **c** Structural classification of compounds on the ESI-MS spectra for PS-12min as a function of ion mass/charge number (m/z). Linear hydrocarbon (Linear HC, black), polystyrene (PS, red), hydrogenated polystyrene (Hydro-PS, blue), and dehydrogenated polystyrene (Dehydro-PS, green) were identified and represented. **d** Comparison of carbon number distribution among PS-2min, PS-6min, and PS-12min.

Detailed structural and compositional information of the liquid products was obtained with Matrix-assisted Laser Desorption/Ionization (MALDI-MS) and Electrospray Ionization (ESI-MS) techniques (“Methods”, Supplementary Note 2). The former one was used to characterize large polymer units (>800 Da), and the latter was used to characterize the low Mw range molecules (0–800 Da)^24^. MALDI-MS detects a series of styrene oligomers with 5–45 monomer units (500–5000 Da) consisting of cyclic (red) and linear (blue) structures with 16 Da Mw difference (Fig. 2a, b, Supplementary Fig. 7), depending on whether end groups (–CH_3_ on one end, –H on the other) were available. A sharp increase in the fraction of 5–15 monomer units was observed with a longer reaction time (Supplementary Fig. 8), suggesting an accelerated degree of depolymerization. Four types of compound structures were identified from the ESI results, including linear hydrocarbons, PS, hydro-PS, and dehydro-PS oligomers (Supplementary Figs. 9–14, Fig. 2c). Their relative amounts as a function of reaction time were analyzed based on the ESI signals intensity (Supplementary Fig. 15), with the trends pointing to possible interconversion among these compounds through hydrogenation. With these characterization results, carbon number and ring distribution of the liquid products are summarized in Fig. 2d and Supplementary Fig. 16–21. A longer reaction time leads to the production of lighter and more narrowly distributed liquid products, with more C_6_–C_9_ chemicals in form of linear alkanes and alkylbenzenes being eventually produced after 12 min of hydrogenolysis reaction (Supplementary Fig. 22). Besides the reaction time, other reaction parameters, including H_2_ flow rate (ν_H2_), partial pressure (*P*_H2_), mass of PS (*m*_PS_) and power input (*P*) were also examined and exhibited certain influences on the PS hydrogenolysis properties (Supplementary Note 3, Supplementary Figs. 23–27).

### D_2_ isotope labeling experiments and proposed reaction pathway

**Fig. 3:**
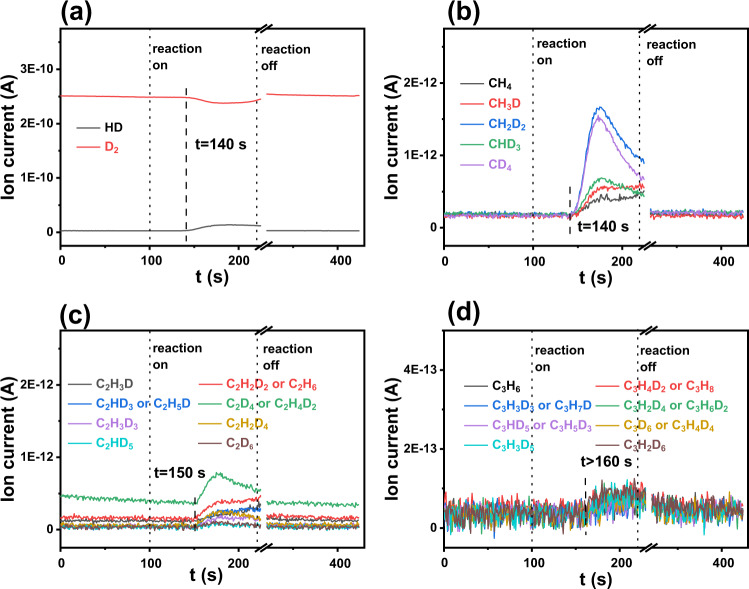
Time-evolved (*t*) gas products evolution during D_2_ isotope labeling PS hydrogenolysis reaction. Mass spectra for (**a**) H_2_ (HD), (**b**) C_1_, (**c**) C_2_, and (**d**) C_3_ species. The reaction was switched on at 100 s, and switched off after 120 s.

**Fig. 4:**
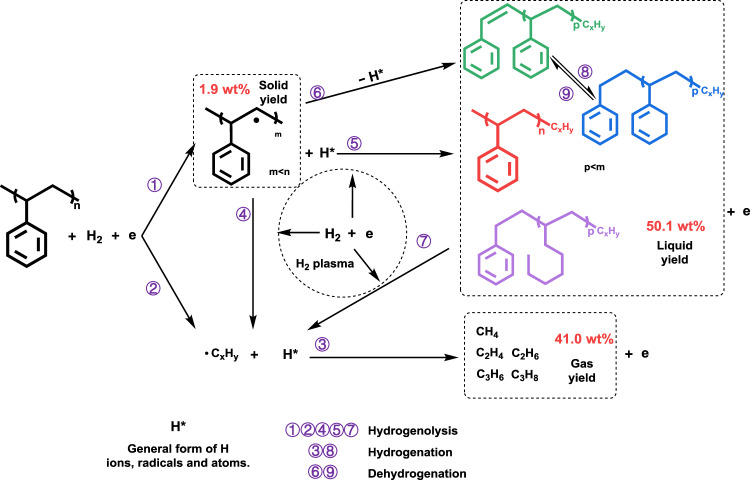
Illustrated reaction pathways for plasma-assisted PS hydrogenolysis. Product yield information shown in the figure is for PS-12min.

D_2_ isotope labeling experiments were conducted by replacing H_2_ with D_2_ to achieve insights into the plasma-assisted PS hydrogenolysis mechanism. It should be noted that the reaction pathway was proposed based on analyses of gas and liquid products, the potential influence of surface etching and irradiation and their impacts on PS decomposition was neglected due to fast kinetics of hydrogenolysis reaction. Figure 3a–d shows the time-evolved MS spectra collected under the reaction condition. HD signals are detected starting from about 40 s after the reaction is switched on (Fig. 3a). Considering only PS contains hydrogen, the generation of HD should be resultant of reaction between highly active deuterium plasma species and PS, which generates hydrocarbon fragments and hydrogen species that would further react with deuterium. Interestingly, the generation of various hydrocarbon gas products exhibits a different reaction time dependence. Methane and deuterium-exchanged methane products begin to be detected simultaneously with HD generation (Fig. 3b), with CH_2_D_2_ and CD_4_ being the major products. In comparison, there are about 10 more seconds of delay for C_2_ products detection and about 20 more seconds of delay for C_3_ products detection (Fig. 3c, d). These results suggest the formation of gas products following an order of CH_4_ > C_2_H_4_ ≈ C_2_H_6_ > C_3_H_6_ ≈ C_3_H_8_ at the initiation of the PS hydrogenolysis. They should be primarily generated via a combination between plasma-activated H species resultant of H_2_ dissociation and •C_*x*_H_*y*_ species resultant of PS fragmentation, either from the PS aliphatic chains or the opened aromatic structures. Based on the collective information of gas and liquid products, a plausible reaction network for the plasma-assisted PS hydrogenolysis is illustrated in Fig. 4. Interacted with H_2_ plasma, C–H and C–C bonds in PS can be effectively broken, resulting in hydrocarbon fragments including smaller units of PS (step 1) and •C_*x*_H_*y*_ species (step 2). The latter would serve as precursors for gas products by means of subsequent hydrogenation (step 3). The former would either be further fragmentized by H_2_ plasma to generate more •C_*x*_H_*y*_ radicals (step 4) and/or form liquid products (PS, hydro-PS) through hydrogenolysis (step 5). Besides, a dehydrogenation pathway to form dehydro-PS would also be likely (step 6), as revealed by the formation of dehydro-PS liquid product (Supplementary Fig. 19) and the HD formation in the isotope labeling experiments (Fig. 3a). Interconversion among liquid products (steps 8 and 9) would occur as well, as evidenced from the time-dependent MS signals (Supplementary Fig. 15).

### Thermodynamic analyses

Thermodynamics of the overall PS hydrogenolysis process was assessed using the Benson group additivity method (Supplementary Note 4, Supplementary Fig. 28 and Supplementary Table 7)^25^. The thermodynamic contributions of individual reactions under the standard condition were calculated and tabulated in Supplementary Table 8. Both depolymerization and gas formation facilitated by plasma-assisted hydrogenolysis, i.e., the major reactions being observed in the experiments, show negative *ΔG* values (Supplementary Table 8), suggesting favorable thermodynamics for the overall reaction under standard condition. All the calculated reactions are exothermic except for the formation of C_2_H_4_ and C_3_H_6_ (Supplementary Table 8). The reactor temperature after 12 min of reaction is measured to be about 200 °C (Supplementary Table 9), which should be resultant of accumulated plasma heat effect and would benefit endothermic C_2_H_4_ formation^26,27^, leading to improvement in kinetics and yields shown in Fig. 1b. Moreover, the net enthalpy that incorporates molar contributions of gas formation reactions (Supplementary Fig. 29) switches the reaction thermodynamics after 2 min of reaction (Supplementary Table 10). The calculated thermodynamic properties, backed up by the measured reaction performance, provide a basis to understand the PS hydrogenolysis process, especially for the formation of gas products.

### Post-consumer PS (PCPS) hydrogenolysis

**Fig. 5:**
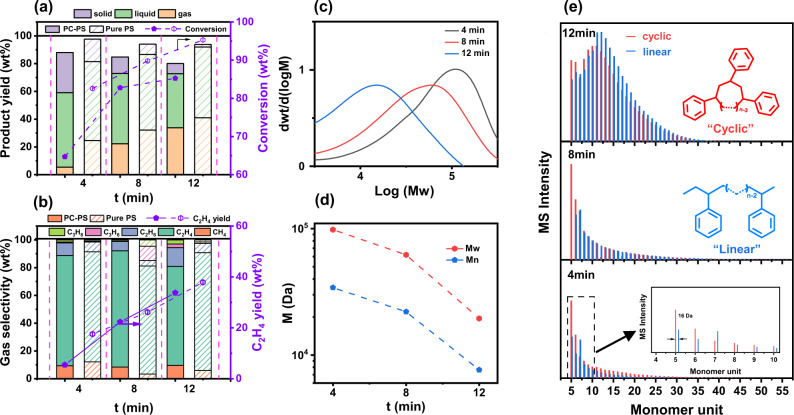
Plasma-assisted post-consumer PS (PCPS) hydrogenolysis properties as a function of reaction time. **a** Product yield and conversion properties. **b** Gas products selectivity and ethylene yield properties. **c**, **d** GPC spectra and molecular weight evolution as a function of reaction time. **e** MALDI-MS spectra of liquid components obtained from PCPS-4min, PCPS-8min and PCPS-12min, respectively. Cyclic and linear polystyrenes were represented in red and blue, respectively.

Our study on post-consumer PS (PCPS) materials proves a good capability of this plasma-assisted hydrogenolysis method to effectively convert PS wastes into valuable chemicals. Commercially available yogurt containers (Supplementary Fig. 30), an important application for PS, were used as feedstock. Identical reaction conditions were applied for PCPS as for pure PS. An 85.2 wt% conversion is achieved after 12 min of reaction (Fig. 5a), which is about 10% lower than that for PS. The existence of unconvertable impurities, such as contaminants, dyes and reinforcement materials, etc., is likely responsible for the conversion reduction by increasing the weight percentage of solid and uncollected substances (Supplementary Tables 11, 12). As high as 41 wt% gas yield is obtained at PCPS-12min, with ethylene still being the dominant gaseous product (71 wt% selectivity and 34 wt% yield, Fig. 5b). The liquid products show a decrease in both yield and average molecular weight as a function of time (Fig. 5a, c, and d). Almost one order of magnitude reduction in the Mw is observed from PCPS-4min to PCPS-12min, based on which a high degree of PS depolymerization from the feedstock can be concluded. This was further confirmed by the MALDI-MS spectra (Fig. 5e). Like the experiments for pure PS, the ESI-MS results exhibit resemblance in the structures of components (Supplementary Figs. 31–34). The carbon number distribution also indicates an enrichment of C_6_–C_9_ chemicals, and a reduction of heavier components with reaction time (Supplementary Fig. 35). Furthermore, the capability of processing various substrates such as polyethylene (PE) and polypropylene (PP), as well as mixtures of the three substrates (3P) was demonstrated (Supplementary Fig. 36). Even higher gas yield was obtained by PE (51.7%), PP (68.5%), and 3P (50.55%) compared with PS, with a dominant gas product varying from plastic monomers. These results suggest the capability of this method in processing real-world plastic wastes which typically consist of mixtures of various polymers.

## Discussion

In summary, our study demonstrates a method for rapid polystyrene hydrogenolysis to light hydrocarbons. Enabled by H_2_ non-thermal plasma, over 90 wt% PS can be swiftly depolymerized and converted to C_1_–C_3_ hydrocarbons and liquid products within 12 min of reaction. The valuable ethylene gas is the most dominating product, accounting for as high as 38 wt% of total yield. Enriched C_6_–C_9_ paraffins and alkylbenzenes that possess high commercial values are obtained in the liquid products. Furthermore, investigation on post-consumer PS materials exhibits similarly efficient gas production regardless of the existence of contaminants and additives. Our studied method for PS hydrogenolysis not only achieves fast and efficient transformation to valuable gas products, but also shows the capability of processing waste plastics with complicated forms and conditions, which is one of the most challenging issues for plastic valorization.

Despite these advantageous features revealing a good potential of this method for real application that helps to realize recycling of currently untouched plastic waste materials, some issues need to be considered before taking on a larger scale. The most challenging problem, not only for the plastic processing but for any other better developed NTP-assisted reactions such as CO_2_ conversion, VOC removal, and N_2_ fixation is the energy efficiency^28,29^. The mismatch between the power input and output (represented by the amount of product) restrains further application of this technique. The time-dependent energy efficiency (η, g/kWh) of gas production is calculated and shown in Supplementary Fig. 3. Methane and ethylene demonstrated the highest energy efficiency between 6 and 7 g/kWh within 4 min of the reaction, and gradually decreases with a longer reaction time. Comparing with other well-established DBD plasma-assisted reactions such as CO_2_ conversion, VOC abatement and N_2_ fixation (Supplementary Fig. 3b), the optimal energy efficiency values obtained from gas products only in the plastic hydrogenolysis exhibit comparable or even superior energy efficiency^30,31^. Considering the unaccounted liquid products, the energy efficiency can be even higher. For future perspective, we propose there are three strategies that can be applied to further improve the energy efficiency. First, elegantly designing DBD plasma generator, reactor, as well as plastic packing methods could help mitigate the power lost to non-plasma zone and unavoidable heat. Second, screening appropriate catalyst, which has been proved in our previous study^16^. Third, as the time-dependent energy efficiency shown in Supplementary Fig. 3a suggests the highest energy efficiency was achieved within the first 2–4 min, which then gradually decreased with a longer reaction time. It is suggested that pulsed or segmental instead of continuous plasma to be applied so that the reaction could remain high EE.

An ideal industrial plastic chemical recycling/upcycling method should be able to process waste plastics swiftly, efficiently, and continuously. Currently, the rapidly growing research efforts in plastic hydrogenolysis mainly focus on depolymerizing various plastics in batch reactors, with pressurized H_2_ atmosphere (20–60 bar), high temperature (200–300 °C) and long reaction time (1–40 h) being applied (Supplementary Table 13). Our presented method outperforms the state-of-art studies in the promise for scaling up with fast, selective, and continuous gas production.

## Methods

### Materials and gases

Polystyrene (PS, Sigma Aldrich, average Mw 35000, Stock No. 331651). Chloroform-D (99.8%, Cambridge Isotope Laboratories, Inc. Lot# DLM-7-PK), CHCl_3_ (>99.8%, Sigma Aldrich, Lot# 132950). H_2_ (99.999%), D_2_ (99.999%) and Ar (99.999%) gases were purchased from Praxair. Calibration gas for 1% methane (CH_4_, 135N-1%), acetylene (C_2_H_2_, 17L-m24-1%-CGD), ethylene (C_2_H_4_, 17L-62a-1%), ethane (C_2_H_6_, 17L-m23-1%), propylene (C_3_H_6_, 17L-293N-1) and propane (C_3_H_8_, 17L-175-1) were purchased from CAL GAS DIRECT^TM^.

### Plasma-assisted PS hydrogenolysis reaction

Non-thermal plasma-assisted PS hydrogenolysis experiments were conducted in a quartz tube reactor equipped with a dielectric barrier discharge (DBD) plasma generator, with cold hydrogen plasma being generated at the plasma generation zone (Supplementary Fig. 1). The volume of the plasma zone (*V*_plasma_) is approximately 6.28 cm^3^. In a typical run, 200 mg PS pellets (sieved to the size of 125–250 µm) were packed in the DBD reactor. The standard reaction condition was100 ml/min H_2_ flow rate, 101 kPa H_2_ partial pressure and 90 W plasma power unless being stated otherwise. Influences of various reaction parameters, including reaction time (2–12 min), H_2_ flow rate (20–100 ml/min), H_2_ partial pressure (0–101 kPa), mass of reactant (100–600 mg) were examined.

The mass of the gas, liquid, and solid products were mainly measured via weight reduction method. The gas product was quantified by measuring the weight loss of the packed reactor before and after the reaction. The liquid product was obtained from the extractant of CHCl_3_ from the liquid–solid mixture in the reactor, while the insoluble resultants were the solid residues (PS + quartz wool). The weight of the solid product was calculated by the difference between quartz wool and solid residues, and liquid product was calculated by the total weight of CHCl_3_ solution minus the weight of solvent. In Supplementary Table 1 a rigorous mass balance calculation is shown which indicates 1.2% error in terms of PS weight. All experiments exhibit <5% error (Supplementary Table 2). The calculation of conversion (*X*, %) and yield (*Y*, wt%) is shown in the following equations, in which *m*_PS_, *m*_gas_, *m*_liquid_ and *m*_solid_ represent the mass of polystyrene, gas, liquid, and solid products, respectively. *m*_us_ represents the uncollected solid which is not taken into account any one of the product category:

1 $${{{{{\rm{Conversion}}}}}}\,(X)=\frac{{m}_{{{{{{\rm{PS}}}}}}}-{m}_{{{{{{\rm{solid}}}}}}}-{m}_{{{{{{\rm{us}}}}}}}}{{m}_{{{{{{\rm{PS}}}}}}}}\times 100 \%$$

2 $${{{{{\rm{Yield}}}}}}\,\,(Y)=\frac{{m}_{{{{{{\rm{gas}}}}}}}/{m}_{{{{{{\rm{liquid}}}}}}}/{m}_{{{{{{\rm{solid}}}}}}}}{{m}_{{{{{{\rm{PS}}}}}}}}{m}_{{{{{{\rm{solid}}}}}}}\times 100 \%$$

### Product analysis and characterization

Online gas product analysis. The gas effluent products, including CH_4_, C_2_H_2_, C_2_H_4_, C_3_H_6_, and C_3_H_8_, were analyzed with an online Agilent 6890 GC-MS equipped with the automatic gas injector. The gases were quantified separately by externally feeding standard calibration gas. Calibration curves at 0–5% v/v% were obtained for each gas. Selectivity was calculated by the respective gas production rate divided by the overall production rate (g/min).

NMR spectroscopy. Liquid products after plasma-assisted hydrogenolysis reaction were analyzed by ^1^H-NMR and ^13^C-NMR, respectively. ^1^H-NMR experiments were conducted with a Varian Mercury 300 MHz spectrometer. ^13^C-NMR spectra were recorded with Varian NMRS 500 MHz spectrometer. Chemical shifts (*δ*, ppm) were calibrated using residual proton signals of the solvent and referenced to tetramethylsilane (TMS).

Matrix-assisted laser desorption/ionization mass spectroscopy (MALDI-MS). MALDI-MS analyses were conducted with a Bruker UltraflexIII MALDI-ToF/ToF instrument. A sandwich method for matrix and sample application was applied. First layer: 20 mg/ml trans-2-[3-(4-tert-Butylphenyl)-2-methyl-2-propenylidene] malononitrile (DCTB) matrix and10 mg/ml silver trifluoroacetate (AgTFA) salt (10:1). Second layer: tested sample at a given concentration. Third layer: 20 mg/ml DCTB matrix and 10 mg/ml AgTFA salt (10:1). The analyses were performed in reflection and a linear mode. Detection range between 500 and 10,000 Da. Laser intensity was 40%.

Electrospray ionization mass spectroscopy (ESI-MS). The experiments were conducted using Waters Synapt G1 ESI-Q Time-of-flight (ToF) MS instrument. The desolvation temperature was 250 °C, the source temperature was 120 °C, the capillary voltage was 3.00 kV and the flow rate was 10 μl/min. Samples were diluted to 10 μg/ml in methanol.

Gel permeation chromatography (GPC). Analyses were performed on a Waters GPC system comprised of a Waters 1515 isocratic HPLC pump, using two 7.8 × 300 mm Styragel HR 4E THF (waters WAT044240), with a Waters 2414 refractive index detector. The system was calibrated using 14 polystyrene standards ranging 201–411,000 Da. The injection volume was 100 uL with a flow rate of 1.0 mL/min. Samples were diluted in THF in concentrations 1–10 mg/ml. Data were processed using the Waters Breeze™ V3.30 software.

FT-IR spectroscopy. Solid residues (mixture of quartz wool and reacted polystyrene solids) obtained after reaction were characterized by ex situ Fourier-transform IR spectroscopy with Nicolet 6700 IR spectrometer in diffuse reflectance IR Fourier-transform spectroscopy (DRIFTS) mode under ambient condition. The spectra were recorded by collecting 32 scans with a resolution of 2 cm^−1^. No specific correction was applied.

^13^C Solid-state NMR spectroscopy. ^13^C solid-state NMR experiments on pure and reacted PS samples were conducted by a Bruker Avance Ultrashield 300 NMR apparatus equipped with a 4 mm double resonance VT CP/MAS probe. The ^13^C and ^1^H carrier frequencies were 75.6 and 300.1 MHz, respectively. Samples were packed in a 4 mm Zirconia MAS rotor with a Kel-F drive cap and were measured with a MAS speed of 13000 ± 5 Hz at 25 °C. ^13^C and ^1^H rf filed strength was adjusted to 55.6 and 75 kHz, respectively. ^1^H TPPM decoupling was used during acquisition. Cross polarization and recycle delay were set to 2 ms and 3 s, respectively. ^13^C chemical shift was externally referenced as the CH carbon of adamantane as 29.46 ppm.

### D_2_ isotope-labeled plasma-assisted PS hydrogenolysis reaction

The D_2_-exchanged PS hydrogenolysis reaction was conducted in the same tube reactor described in the previous context, connected with a Pfeiffer Omnistar GSD 320 MS instrument. In a typical run, 200 mg PS was packed in the tube reactor, with 100 ml/min D_2_ gas being continuously flowed through, plasma power was set at 90 W for a 2 min reaction.

### Post-consumer PS hydrogenolysis

Post-consumer polystyrene plasma-assisted hydrogenolysis reaction was conducted using commercially available yogurt packages that are made of polystyrene blended with dyes and reinforcement materials. The post-consumer material was cut, shredded, and packed in the DBD reactor with a similar configuration to that of pure PS powder. The methods applied for reaction evaluation (i.e. yield calculation, product analysis, and reaction parameters) were identical to those conducted for pure PS material.

## Supplementary information

Supplementary Information

## Data Availability

The main data supporting the findings in this study are provided in the paper and Supplementary information. Additional data are available from the corresponding authors upon reasonable request.
