# Supplementary material for: Non-thermal plasma-assisted rapid hydrogenolysis of polystyrene to high yield ethylene
Source: Nat Commun. 2022 Feb 16;13:885. doi: 10.1038/s41467-022-28563-7 (PMC8850602; doi:10.1038/s41467-022-28563-7)
Supplement: Supplementary file 1 — Supplementary Information [file 41467_2022_28563_MOESM1_ESM.pdf]

Supplementary Information for

Non-thermal Plasma-assisted Rapid Hydrogenolysis of Polystyrene to High Yield Ethylene

Libo Yao,[1] Jaelynne King,[2] Dezhen Wu,[1] Jiayang Ma,[2] Jialu Li,[1] Rongxuan Xie,[1] Steven S.C. Chuang,[2] Toshikazu Miyoshi,[2] and Zhenmeng Peng[1]*

[1]Department of Chemical, Biomolecular and Corrosion Engineering, the University of Akron, 200 E Buchtel Avenue, Akron, OH 44325, United States.

[2]School of Polymer Science and Polymer Engineering, The University of Akron, 170 University Avenue, Akron, OH 44325, United States.

*Correspondence to: zpeng@uakron.edu

Table of Contents:

Supplementary Notes 1–4
Supplementary Fig.s 1–36
Supplementary Tables 1–13
Supplementary references

Supplementary Notes

Supplementary Note 1: Mass balance calculations

For the terms involved in the calculation, m_{qw} and m_{PS} are mass of quartz wool and PS, respectively. The terms reactor before (m_{rb}), reactor after (m_{ra}) and reactor empty (m_{re}) correspond to the weight of packed reactor before reaction, right after reaction and reactor when all the reactants were removed, respectively. Uncollected solids (m_{us}) represent the solid materials that are clung to the surface of the tube reactor or electrode. m_{CF} and m_{ex} are weight of chloroform solvent and the total weight of $CHCl_3$ soluble solution, respectively. Filtered solids (m_{fs}) indicates the filtered insoluble substance from the $CHCl_3$ extractants. m_{sr} refers to the total weight of chloroform insoluble substances. It should be noted that m_{us} and m_{fs} were not counted into any of gas, liquid or solid products as their composition cannot be determined.

Supplementary Note 2: MALDI- and ESI-MS spectra for liquid products of PS hydrogenolysis

The MALDI spectra shows distributions of PS oligomers with Mw ranging from 500–5000 Da. In the time-dependent trend, sharply increased signal intensity for PS species with 5–15 monomer units was observed. This indicates accelerated degree of depolymerization as increase of reaction time. Meanwhile, a general trend for transformation from cyclic to linear structure can also be witnessed.

Supplementary Fig. 9–13 show time-evolved compound structures classified by the four categories based on ESI-MS results, and Supplementary Fig. 14 shows the summary in terms of MS intensity in a time-dependent manner. It can be observed that linear hydrocarbons present monotonous increase, while dehydro-PS shows monotonous decrease. PS and hydro-PS species exhibit V and reverse V trend as a function of time, respectively. The overall evolution for different species gives a hint about reactions for liquid components. Linear hydrocarbons can be derived from direct cleavage of PS chains, or from the opened aromatic rings. Its increase is a direct evidence for deepened degree of depolymerization, which is also evidenced by decrease for dehydro-PS that is most likely obtained from the dehydrogenation reaction facilitated by plasma bombardment. As for PS and hydro-PS, the reverse trend shows conversion from PS to hydro-PS as reaction proceeds, causing monotonous decrease for PS and increase for hydro-PS from 0–6 min. PS bounces back from PS-8min, which is possibly caused by depolymerization of more solid products. Meanwhile hydro-PS turns downward as a possible resultant of ring opening and cleavage due to a deepened degree of hydrogenation, which turns itself into PS species. Therefore, based on the assumption that the MS counts is relatively reliable to represent the abundance of each species with absence of rigorous calibration of each compound, the inter-conversion network among these species is proposed. PS and dehydro-PS are resultant of plasma bombardment/hydrogenolysis, while hydro-PS is the hydrogenation products from the previous species. PS can also be formed via dehydro-PS or hydro-PS hydrogenation. Linear hydrocarbons can be

formed through chain cleavage from other three species. Such understanding is used for deriving the reaction pathway.

Supplementary Note 3: Reaction parameters on PS hydrogenolysis performance

Evaluation of ν_{H2} (0–100 ml/min) on PS hydrogenolysis reaction performance is conducted and the results are shown in Supplementary Fig. 22 and Supplementary Table 4. Product distribution indicates less than half of gas products were yielded in 20 ml/min compared with that of 100 ml/min (Supplementary Fig. 22b, with ethylene still being the dominant gas species for all conditions (S_{C2H4} > 70 wt%). Liquid component takes up as high as > 70 wt% of total yield when ν_{H2}=20 ml/min, suggesting low H_2 flow rate is advantageous to liquid yield. Such conclusion is also corroborated by GPC results in Supplementary Table S4, from which the Mw at 20 ml/min was reduced by a factor of 3 in comparison to that of 100 ml/min (high Mw mode). Therefore, it can be concluded lower ν_{H2} benefits favors liquefaction of PS, while higher ν_{H2} favors gas production. The difference between the reaction behavior is likely caused by density of H_2 plasma.

The influence of H_2 partial pressure (0–101 kPa, balanced by Ar) was also investigated, increasing P_{H2} shows positive influence on hydrogenolysis performance, leading to enhanced conversion and gas/liquid yield (Supplementary Fig. 23). It’s worth noted that pure Ar results in minimal PS conversion, especially in terms of gas formation (1.6 wt% yield, Supplementary Table 5), indicating H_2 plasma (in the form of ions, radicals and dissociated atoms) is essential in depolymerizing PS structure.

Investigation on mass of reactant, which was used to represent efficiency of energy input, witnesses peaked conversion and gas production (91.2 wt% and 58.9 wt%, Supplementary Fig. 24) with 100 mg PS, the values of which decay with larger amount of PS (up to 600 mg), accomponied by elevated solid fractions (Supplementary Table 6). Despite smaller reactant benefits more thorough hydrogenolysis, it also causes excessive energy consumption. Therefore, the suitable amount of PS used for each run should be between 200–400 mg.

Investigation on power input suggests higher reactivity (gas yield, conversion) was achieved with higher power (Supplementary Fig. 27) , i.e. greater plasma intensity, suggesting more intense hydrogenolysis reaction. However, higher power input could also incur greater energy loss, as can be observed from Supplemetary Fig. 27c. The energy efficiency normalized by gas weight plateaued between 90–120 W, and decreased upon higher value.

Supplementary Note 4: Thermodynamic calculations using Benson group additivity

The incremental thermodynamic properties were calculated based on Benson group additivity,[1-3] with the values of individual group listed on Supplementary Table 7. In order to calculate the additive properties of polystyrene, the molecule ($C_{8n}H_{8n}$+16, with end groups on both end) was broken down into 5 groups (Supplementary Table 7) so that the overall thermodynamic contributions of ($C_{8n}H_{8n}$+16) can be represented below in Eq. S1:

$$m(C_{8n}H_{8n} + 16) = n(6m_1 + 5m_2 + m_3 + m_4) + m_5 \qquad \text{(Eq. S1)}$$

Based on the analyses of the products, the thermodynamic properties of

hydrogenolysis reaction of polystyrene can be represented by the change of additive terms.[5] In Supplementary Fig. 29, the proposed reaction schemes for chain cleavage and methane formation are provided. The thermodynamic contributions of both reactions are calculated following the proposed scheme as example demonstration. In Eq. S2, m_{depoly} is termed as thermodynamic property (H, S, G…) for the depolymerization reaction, the overall reaction results in increase of one m_5 group, and reduction of one m_3 and m_6 groups, therefore, m_{depoly} is written in the following:

$$m_{depoly} = -m_4 - m_5 + m_6 \qquad \text{(Eq. S2)}$$

Likewise, thermodynamic contributions for methane formation reaction m_{CH4} can be represented by:

$$m_{CH4} = m_4 + m_7 - 2m_3 - 2m_6 \qquad \text{(Eq. S3)}$$

The overall contributions of gas formations (molar basis) were summarized in a time-dependent manner (Supplementary Table 9). All reactions exhibit negative Gibbs free energy, suggesting spontaneity for the overall hydrogenolysis reaction. Enthalpy (ΔH) turns to positive value between 2 min and 4 min reaction, and remains positive with longer reaction time, indicating transfer from exothermic to endothermic for the overall gas formations. Investigation on inevitable heat effect[4-6] of the H_2 plasma was conducted by measuring the temperature of PS bed after reaction, which shows rapid temperature increase within first 4 min from room temperature to ~ 200 ℃, and remains at this level ever since (Supplementary Table 10). The drastic temperature increase caused by DBD plasma heat effect was accompanied by significant increase for ethylene selectivity (Fig. 1b) Considering ethylene formation is the most endothermic reaction based on the calculation (Supplementary Table 8), this explains the initially high methane selectivity and dominant ethylene selectivity with longer reaction time, as well as the overall endothermic characteristic for gas formations.

Thermodynamic properties of all chain cleavage and gas formation reactions were calculated by applying the same method and are summarized in Supplementary Table 8. It’s worth noting that the reactions for aromatic hydrogenation, the inter-conversion between different categories of compounds were not taken into account as they made little contributions to the overall thermodynamics.[7,8]

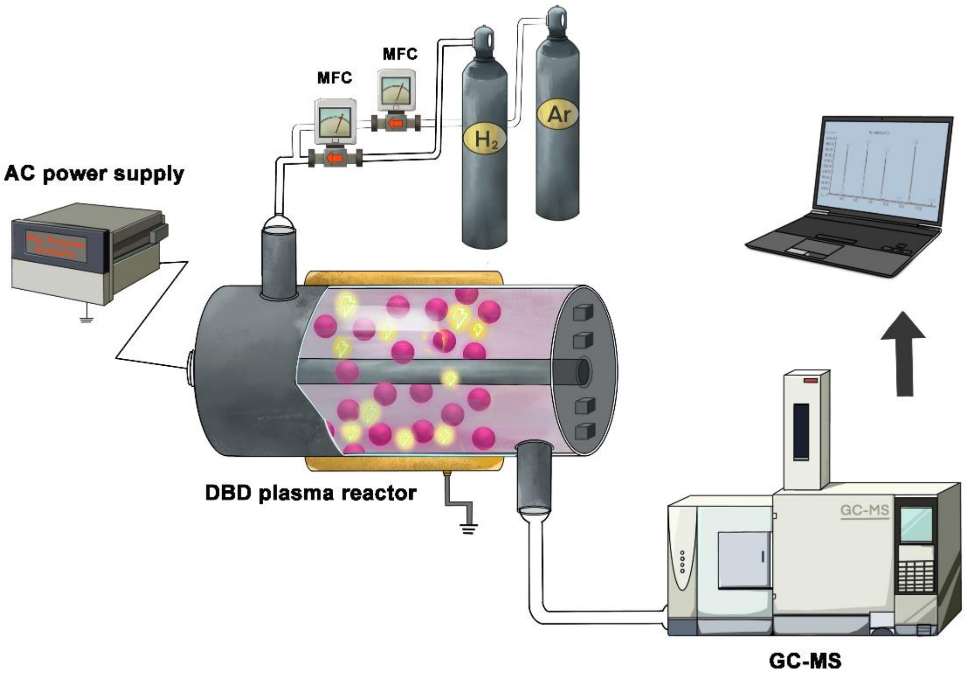

Supplementary Fig. 1. Schematic illustration for the reaction system of DBD plasma-assisted PS hydrogenolysis.

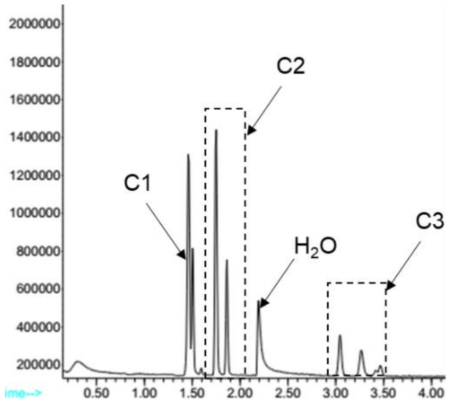

Supplementary Fig. 2. GC-MS spectra of PS hydrogenolysis reaction. The H_2O peak comes from the background, not as product.

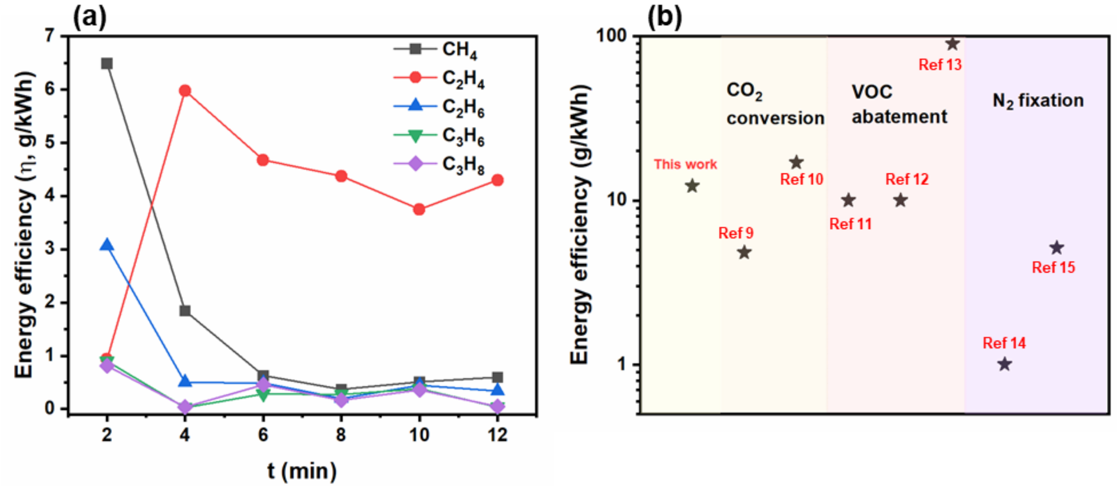

Supplementary Fig. 3. Energy efficiencies (η) of gas products for PS plasma-assisted hydrogenolysis (a) and comparison with other non-thermal plasma-assisted catalytic reactions (b).[9-15] The non-thermal plasma-assisted PS hydrogenolysis achieved as high as 12.2 g/kWh energy efficiency in terms of gas formation only, which is comparable to the values for plasma-assisted CO_2 conversion and VOC removal reactions, and superior efficiency for N2 fixation reaction is observed, suggesting energetic advantage of this method.

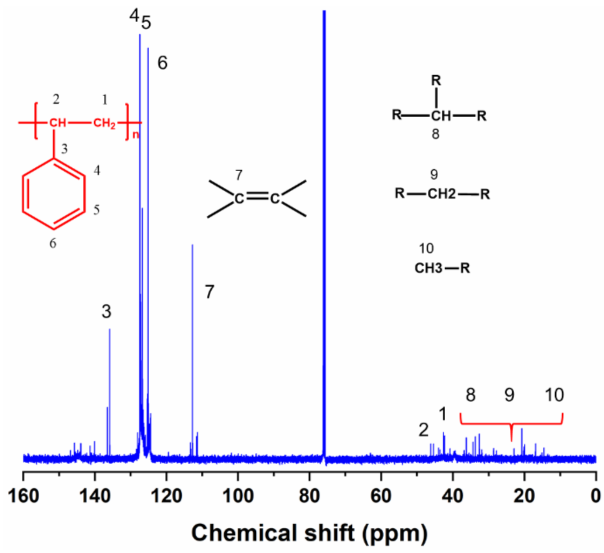

Supplementary Fig. 4. ^{13}C solution-state NMR spectrum of liquid product from PS-6min.

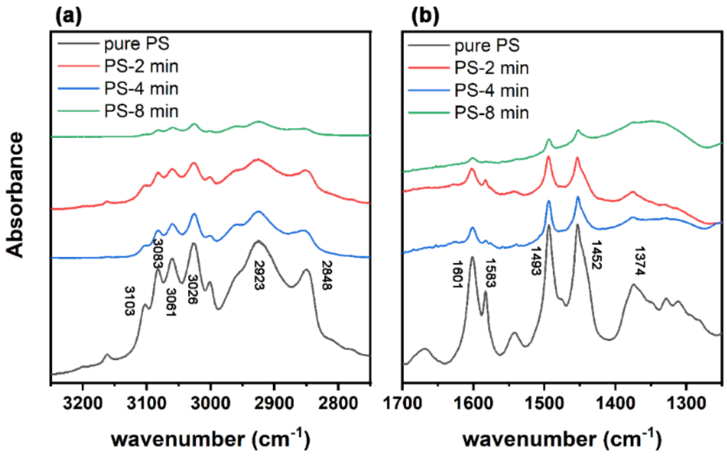

Supplementary Fig. 5. FT-IR spectra of pure and reacted PS samples. The FT-IR spectra for samples before and after hydrogenolysis show decayed but retained signals for characteristic peaks of polystyrene,[16,17] and no new bands were detected. This shows the solid residues remain in the type of polystyrene with reduced quantity.

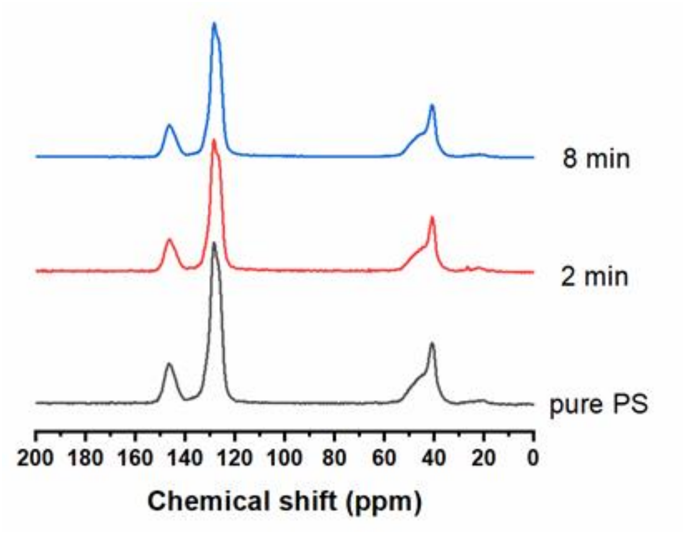

Supplementary Fig. 6. ^{13}C CPMAS NMR spectra for pure and reacted PS samples at 25 °C. The ^{13}C cross polarization magic angle spinning (CP/MAS) NMR spectra suggest no change for the solid materials except for intensity reduction before and after reaction. Combined with the FT-IR results, it is concluded that the solid remnants after hydrogenolysis reaction are still polystyrene.

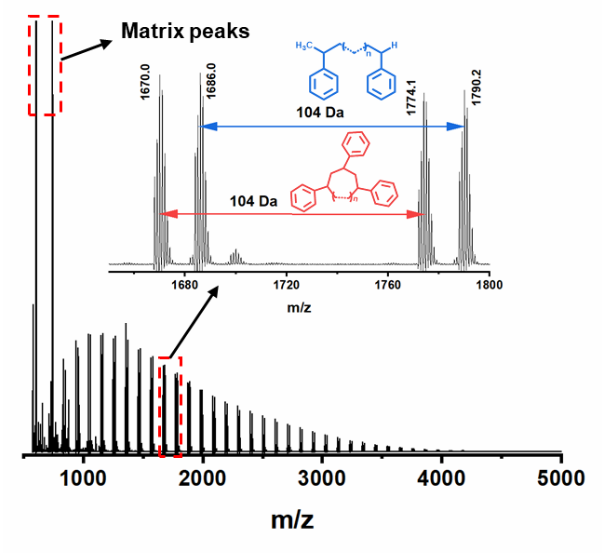

Supplementary Fig. 7. Example of original MALDI-MS spectra for PS-10min. The inset shows two adjacent sets of MALDI signals positioned at 1670-1790 Da. Cyclic and linear PS were identified and indicated. Reaction condition for the obtained sample: v_{H2}=100 ml/min, P_{H2}=101 kPa, t=10 min, P=90 W.

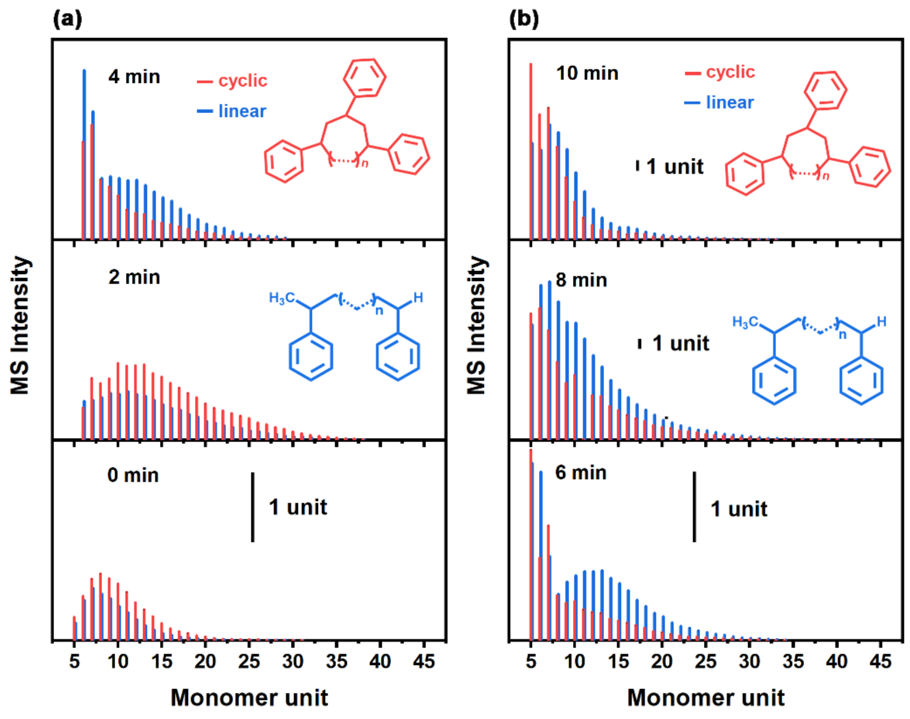

Supplementary Fig. 8. Summary of MALDI-MS spectra for liquid products obtained under increasing reaction time. (**a**) 0-4 min, (**b**) 6-10 min. Spectra presented in terms of number of styrene monomer units (multiples of 104 Da). In (**a**), three spectra were presented in the same scale while in (**b**), two different scales were marked.

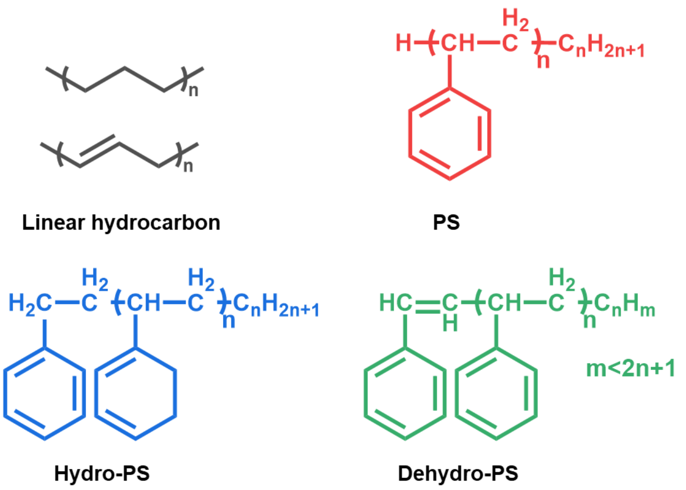

Supplementary Fig. 9. Representative structures of four categories of compounds identified from ESI-MS spectra.

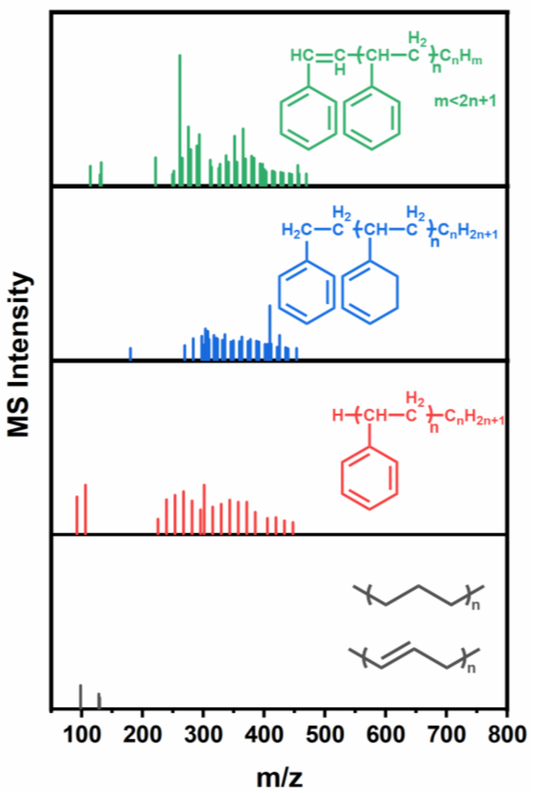

Supplementary Fig. 10. Structural classification of PS-2min liquid product based on ESI-MS spectra (0-800 Da).

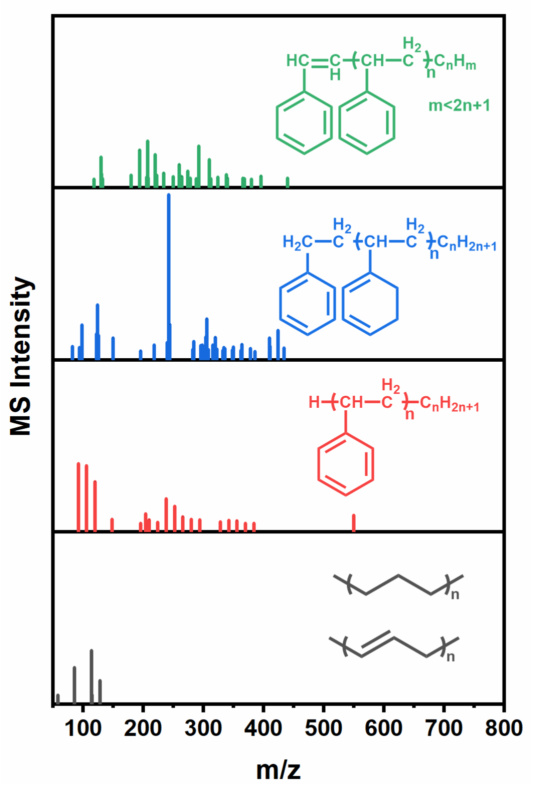

Supplementary Fig. 11. Structural classification of PS-4min liquid product based on ESI-MS spectra (0-800 Da).

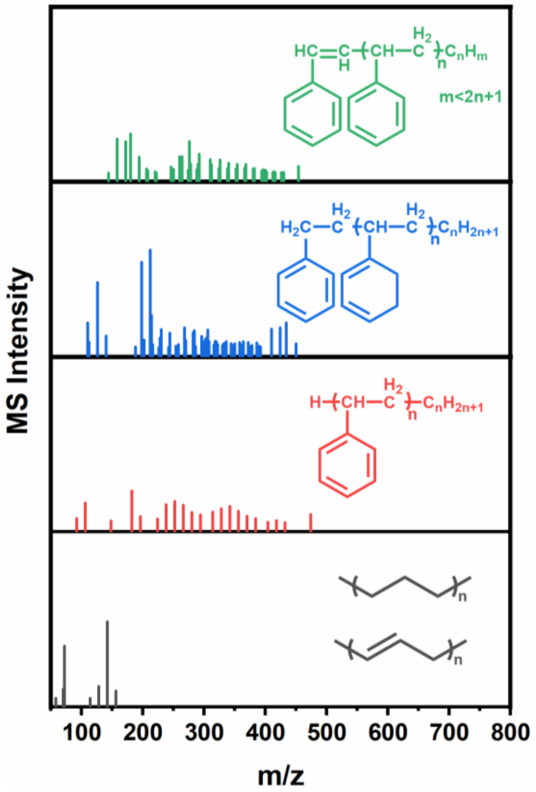

Supplementary Fig. 12. Structural classification of PS-6min liquid product based on ESI-MS spectra (0-800 Da).

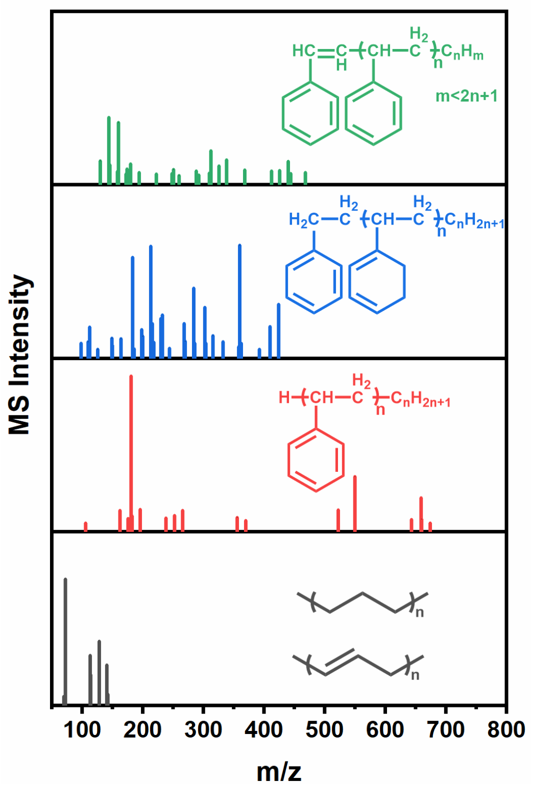

Supplementary Fig. 13. Structural classification of PS-8min liquid product based on ESI-MS spectra (0-800 Da).

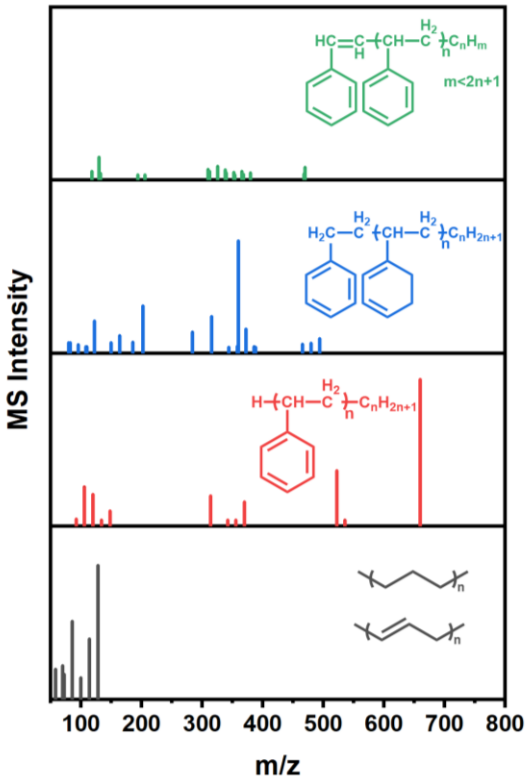

Supplementary Fig. 14. Structural classification of PS-10min liquid product based on ESI-MS spectra (0-800 Da).

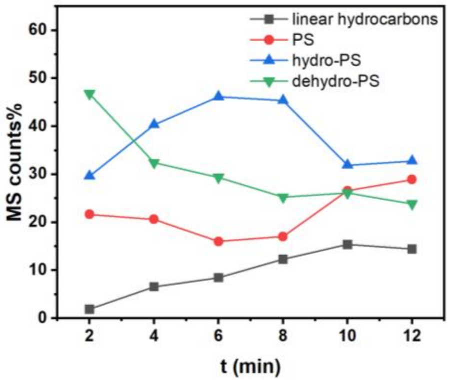

Supplementary Fig. 15. Summarized MS counts percentage for the 4 categories of compounds and their evolution as a function of reaction time.

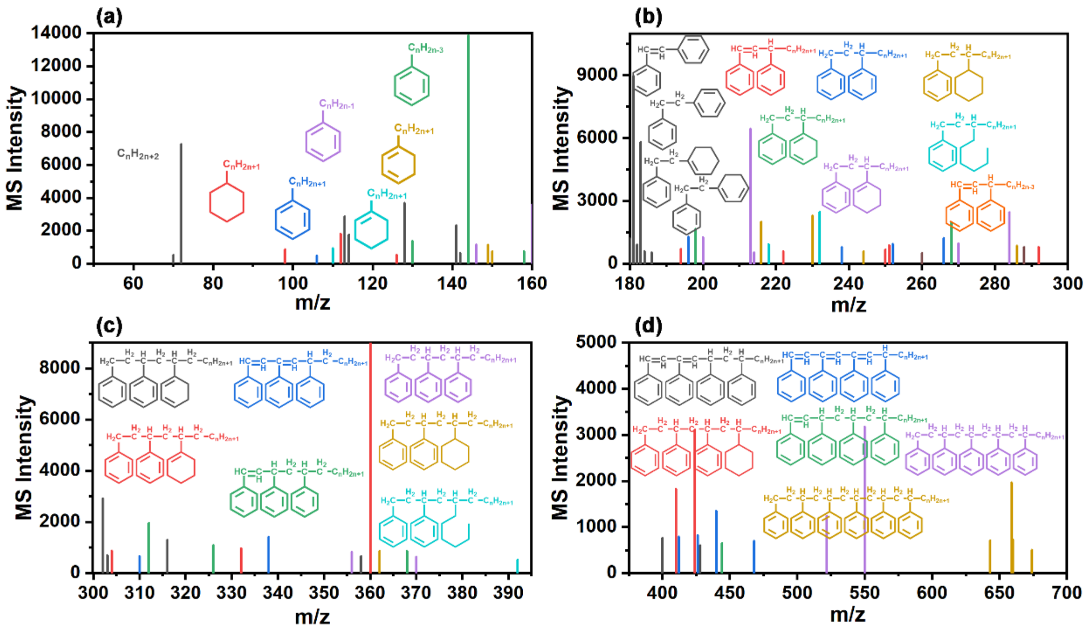

Supplementary Fig. 16. Detailed structural identification for PS-2min ESI-MS result.

Structures classified by number of rings. (**a**) 0-1 ring, (**b**) 2 rings, (**c**) 3 rings and (**d**) 4-6 rings.

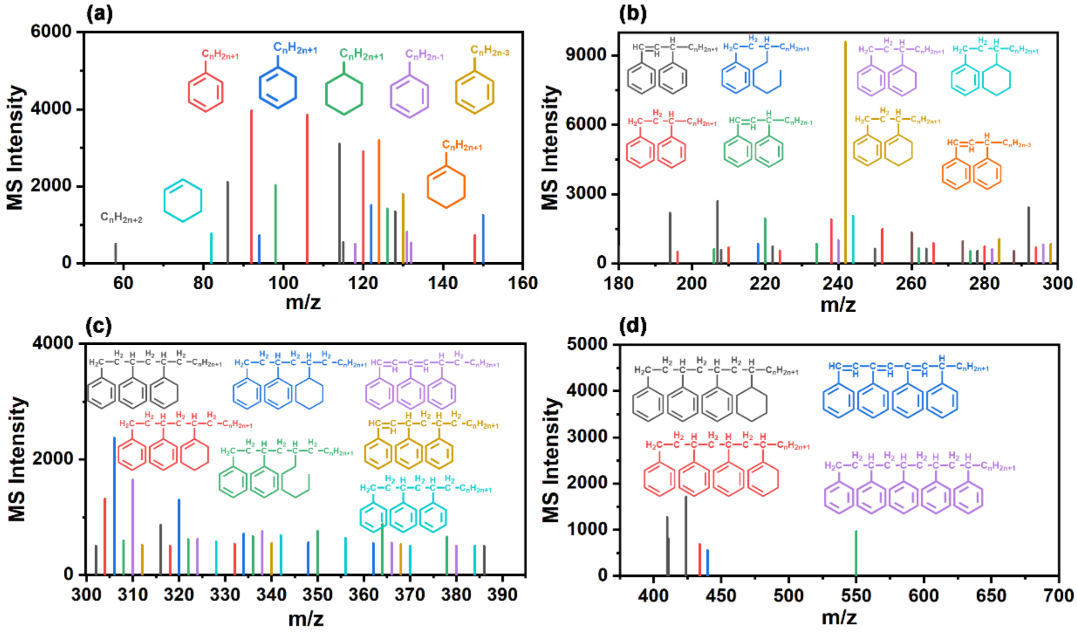

Supplementary Fig. 17. Detailed structural identification for PS-4min ESI-MS result.

Structures classified by number of rings. (**a**) 0-1 ring, (**b**) 2 rings, (**c**) 3 rings and (**d**) 4-6 rings.

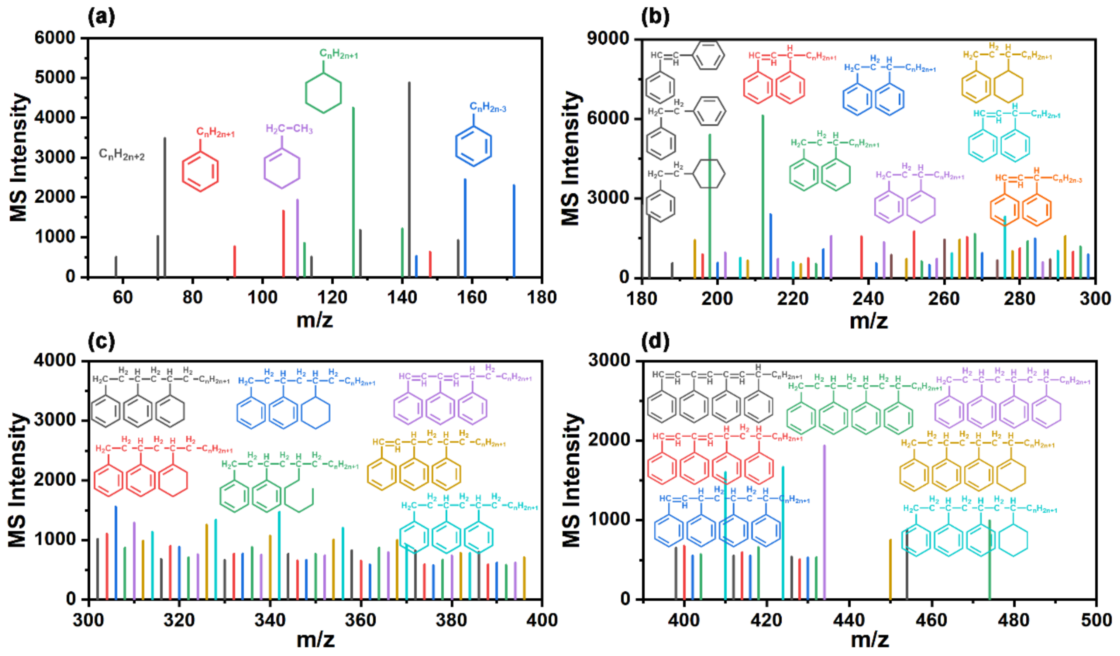

Supplementary Fig. 18. Detailed structural identification for PS-6min ESI-MS result.

Structures classified by number of rings. (**a**) 0-1 ring, (**b**) 2 rings, (**c**) 3 rings and (**d**) 4-6 rings.

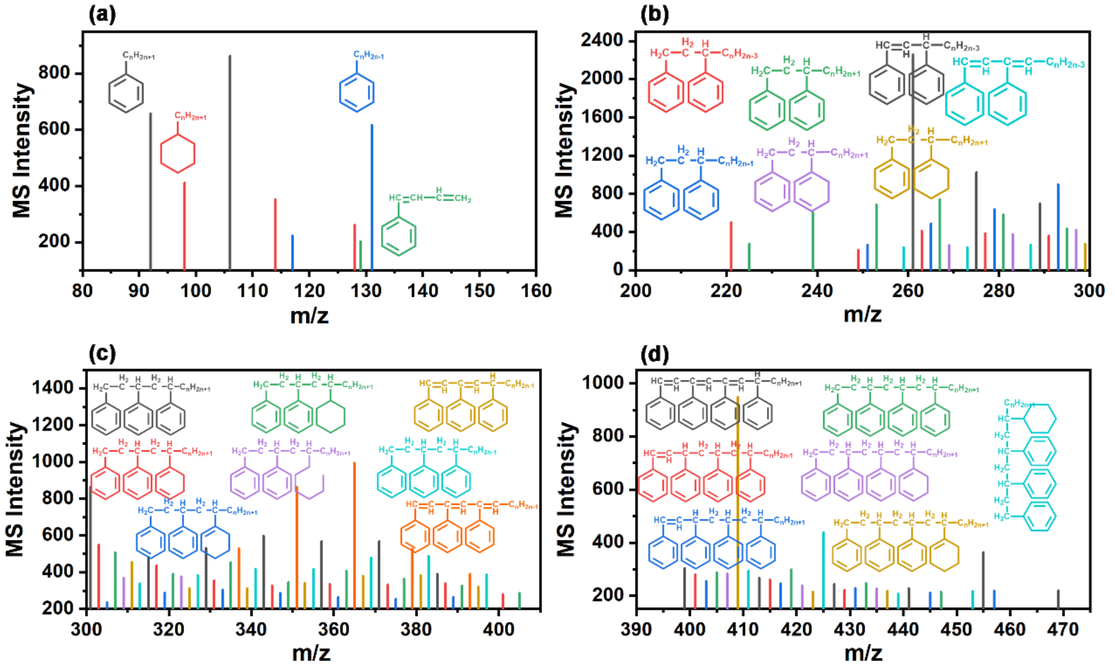

Supplementary Fig. 19. Detailed structural identification for PS-8min ESI-MS result.

Structures classified by number of rings. (**a**) 0-1 ring, (**b**) 2 rings, (**c**) 3 rings and (**d**) 4-6 rings.

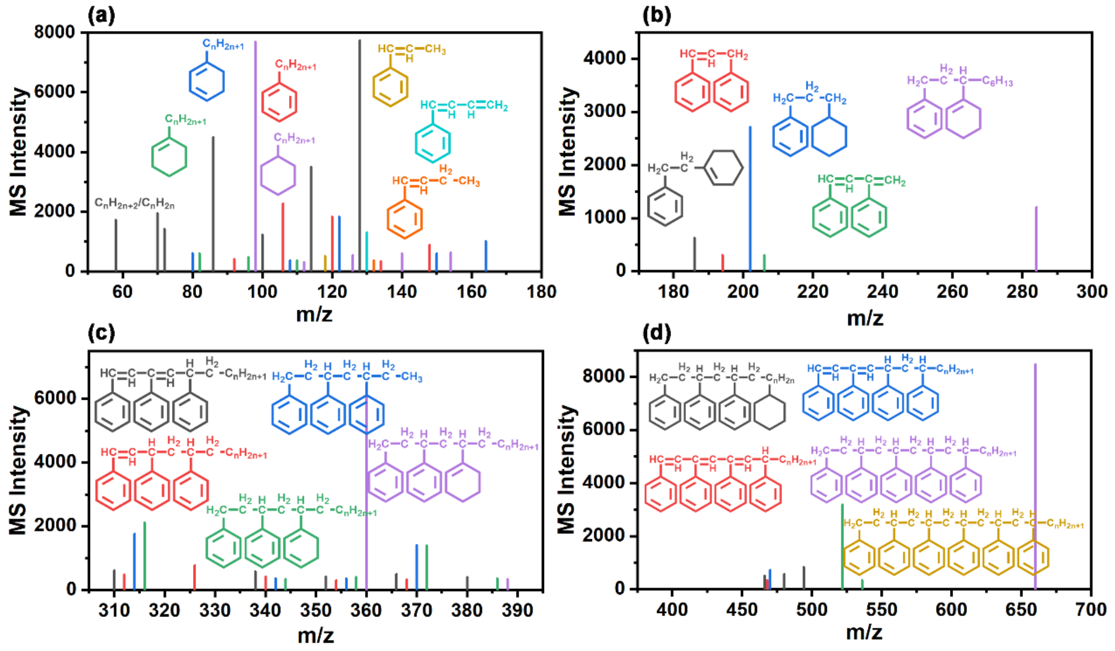

Supplementary Fig. 20. Detailed structural identification for PS-10min ESI-MS result.

Structures classified by number of rings. (**a**) 0-1 ring, (**b**) 2 rings, (**c**) 3 rings and (**d**) 4-6 rings.

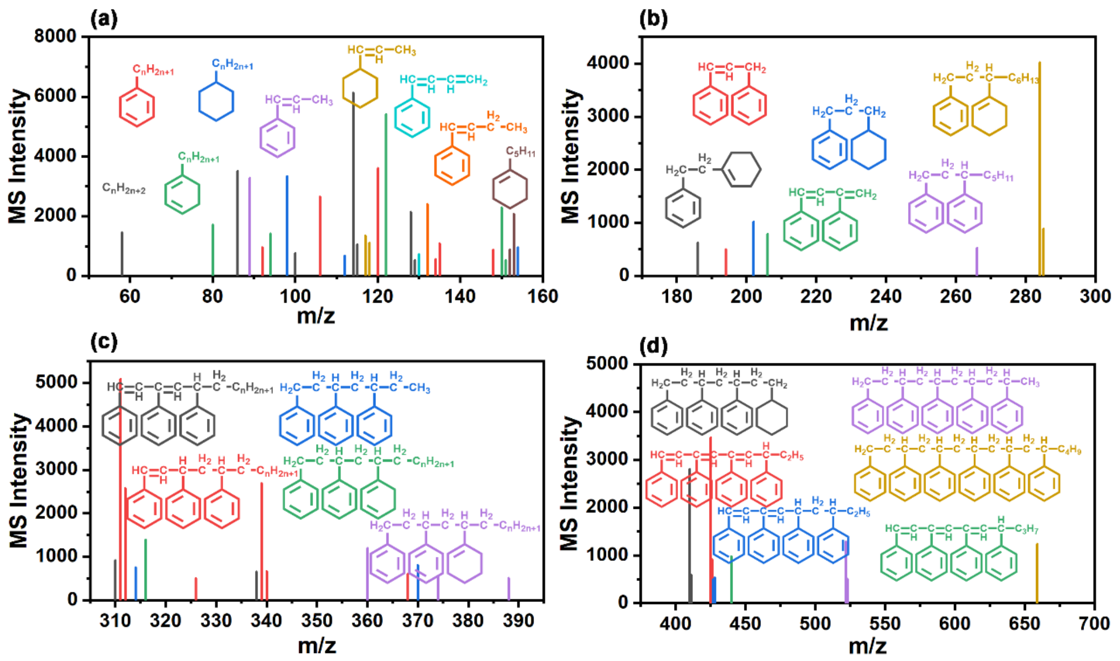

Supplementary Fig. 21. Detailed structural identification for PS-12min ESI-MS result. Structures classified by number of rings. (**a**) 0-1 ring, (**b**) 2 rings, (**c**) 3 rings and (**d**) 4-6 rings.

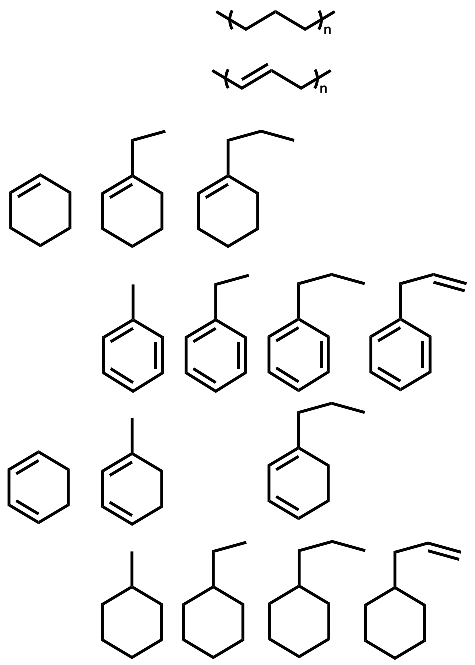

Supplementary Fig. 22. Summary of C_6-C_9 compounds identified from ESI-MS spectra.

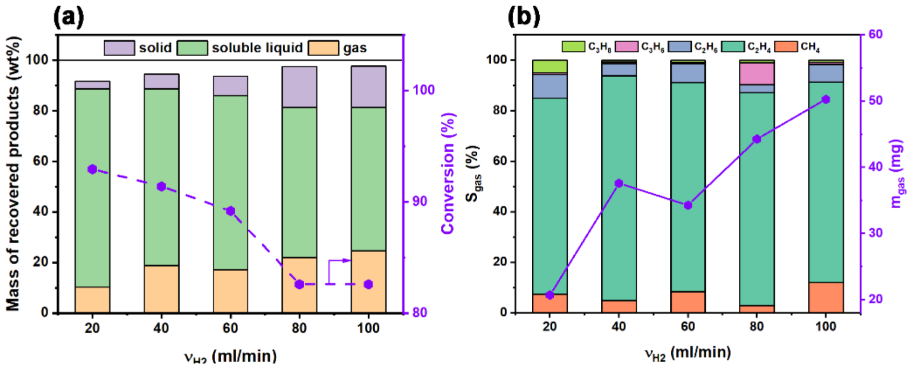

Supplementary Fig. 23. Evaluation of the influence of H_2 flow rate ($ν_{H2}$, ml/min) on the performance of PS hydrogenolysis reaction. Other reaction conditions: t = 4 min, P_{H2} = 101 kPa, m_{PS} = 200 mg and P = 90 W.

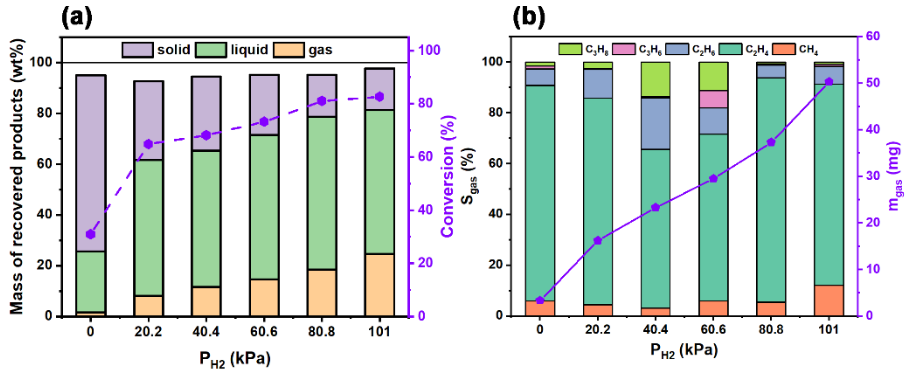

Supplementary Fig. 24. Evaluation of the influence of H_2 partial pressure (P_{H2}, kPa) on the performance of PS hydrogenolysis reaction. Other reaction conditions: t = 4 min, v_{H2} = 100 ml/min, m_{PS} = 200 mg and P = 90 W. H_2 was balanced by Ar.

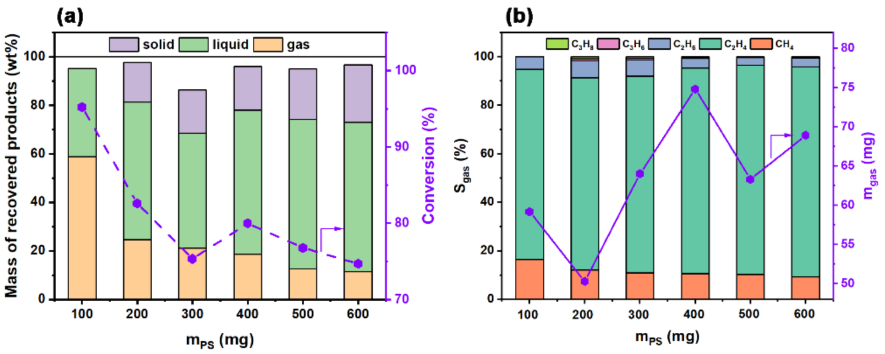

Supplementary Fig. 26. Evaluation of the influence of mass of PS (m_{PS}, mg) on the performance of PS hydrogenolysis reaction. Other reaction conditions: t = 4 min, v_{H2} = 100 ml/min, P_{H2} = 101 kPa, m_{PS} = 200 mg and P = 90 W.

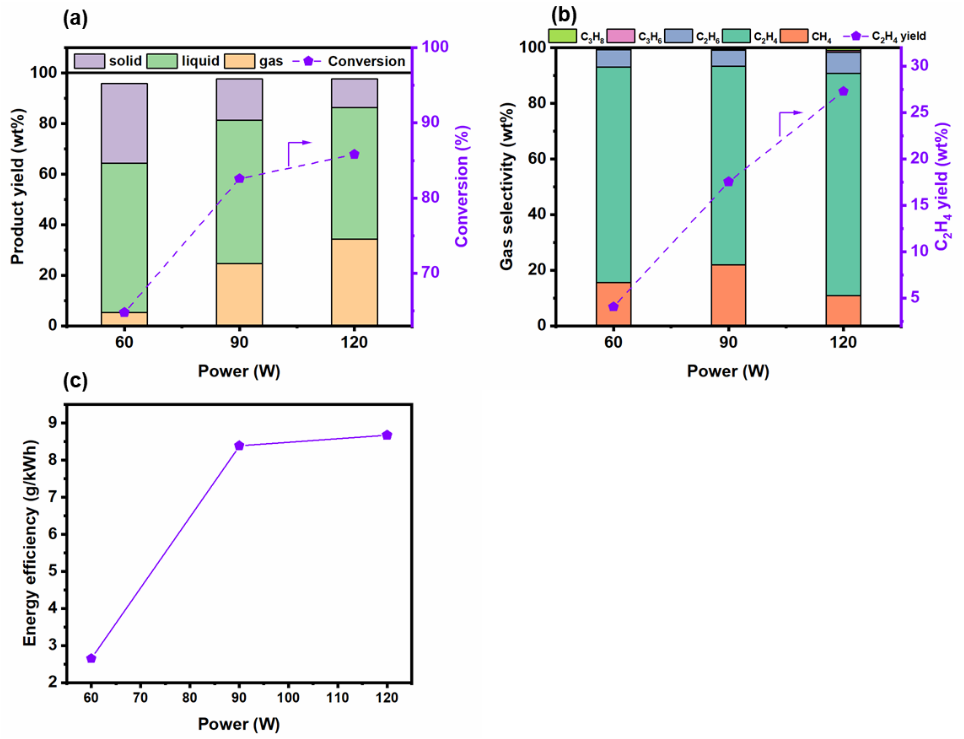

Supplementary Fig. 27. Evaluation of the influence of plasma power on the performance of PS hydrogenolysis reaction. Other reaction conditions: t = 4 min, v_{H2} = 100 ml/min, P_{H2} = 101 kPa, m_{PS} = 200 mg.

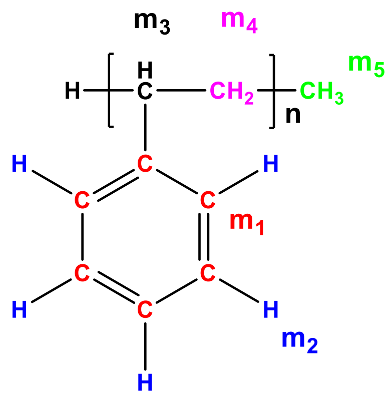

Supplementary Fig. 28. Breakdown of polystyrene additive terms (m_1 – m_5) based on Benson group additivity.

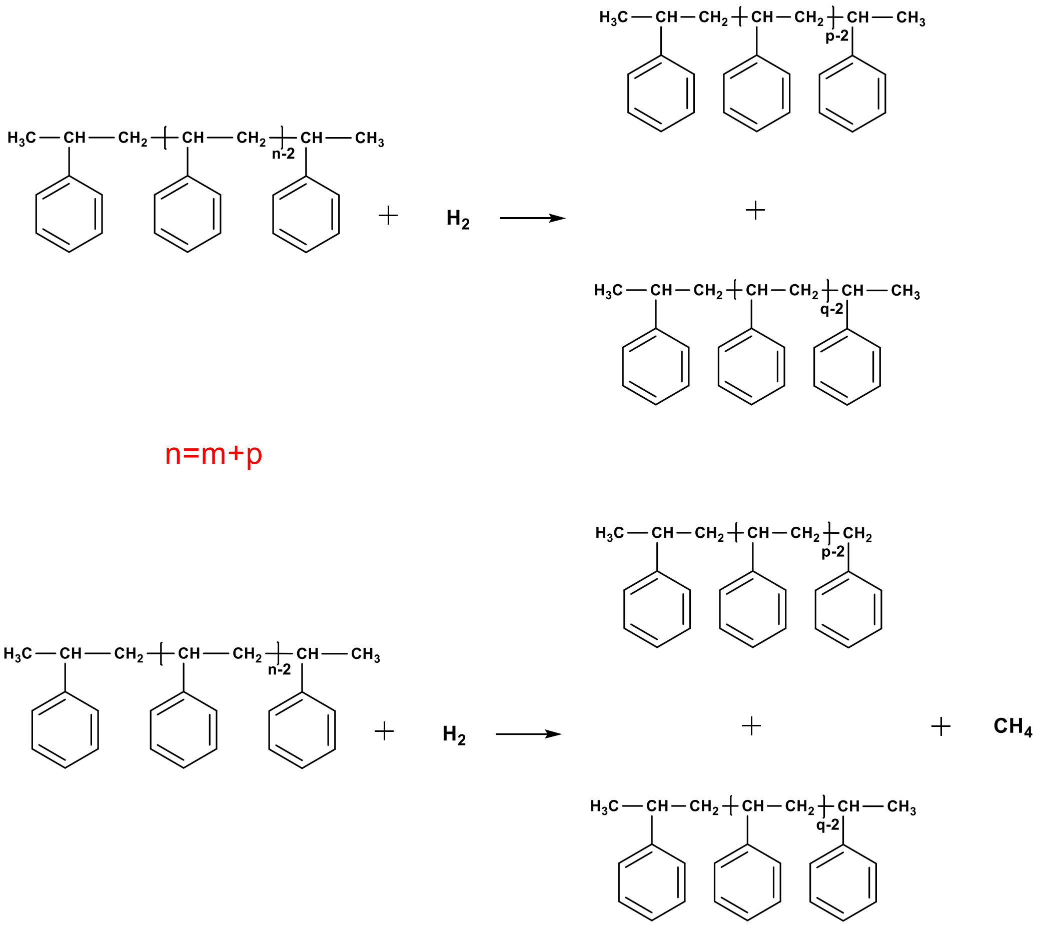

Supplementary Fig. 29. Proposed PS hydorgenolysis reaction scheme for chain cleavage and methane formation.

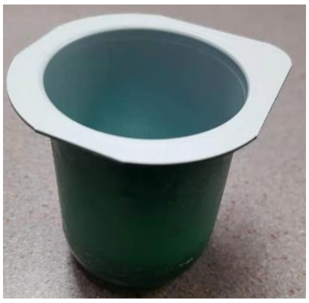

Supplementary Fig. 30. Picture of PCPS material, yogurt package.

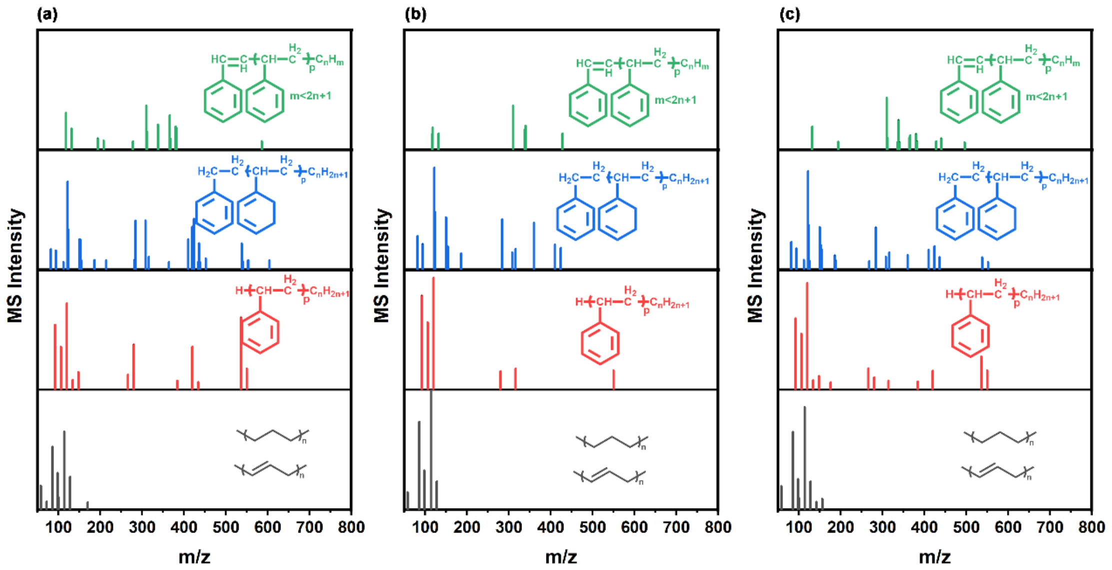

Supplementary Fig. 31. Structural classification based on ESI-MS spectra (0-800 Da) for PCPS hydrogenolysis reaction. (**a**) PCPS-4min, (**b**) PCPS-8min and (**c**) PCPS-12min.

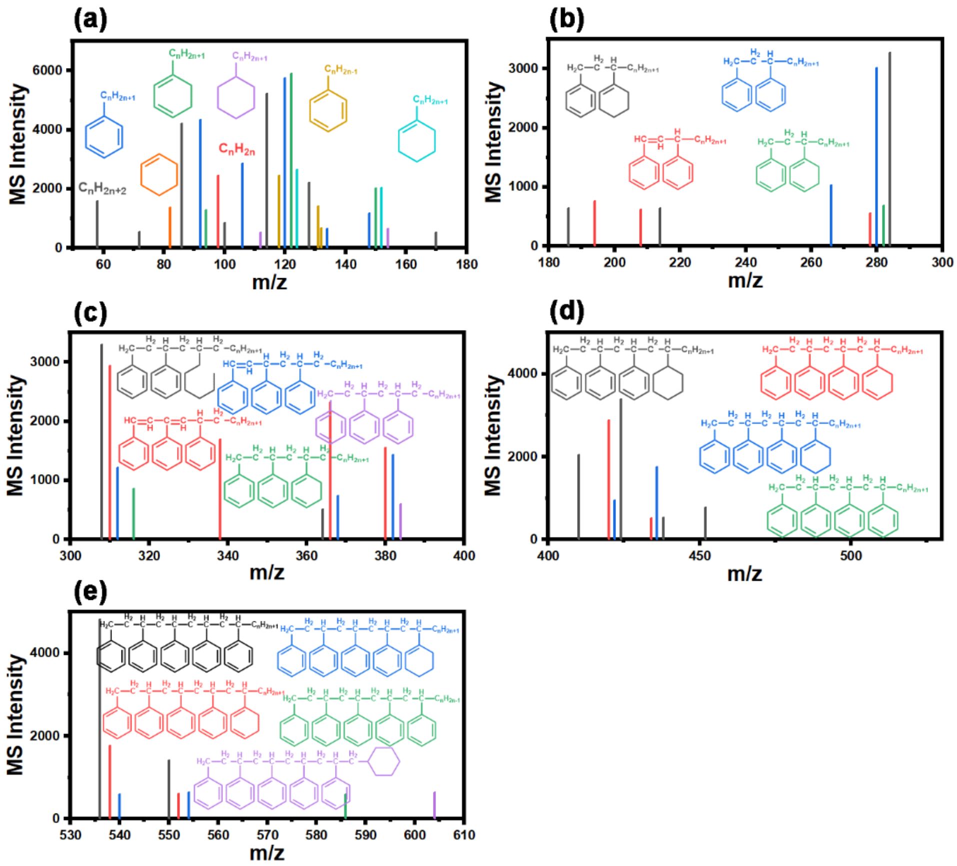

Supplementary Fig. 32. ESI-MS spectra compound identification for PCPS hydrogenolysis.

Reaction condition: v_{H2} = 100 ml/min, P_{H2} = 101 kPa, t = 4 min, P = 90 W.

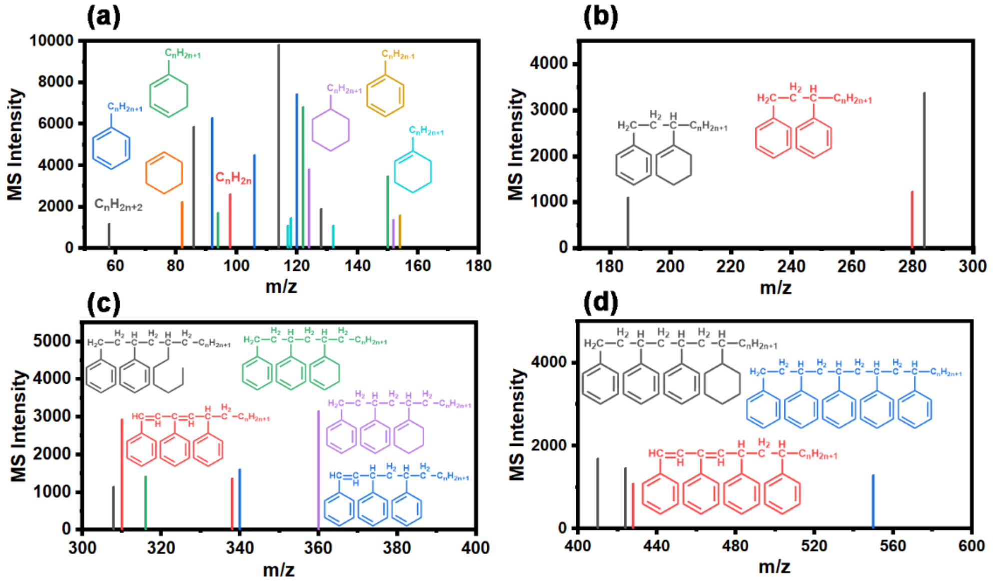

Supplementary Fig. 33. ESI-MS spectra compound identification for PCPS hydrogenolysis.

Reaction condition: v_{H2} = 100 ml/min, P_{H2} = 101 kPa, t = 8 min, P = 90 W.

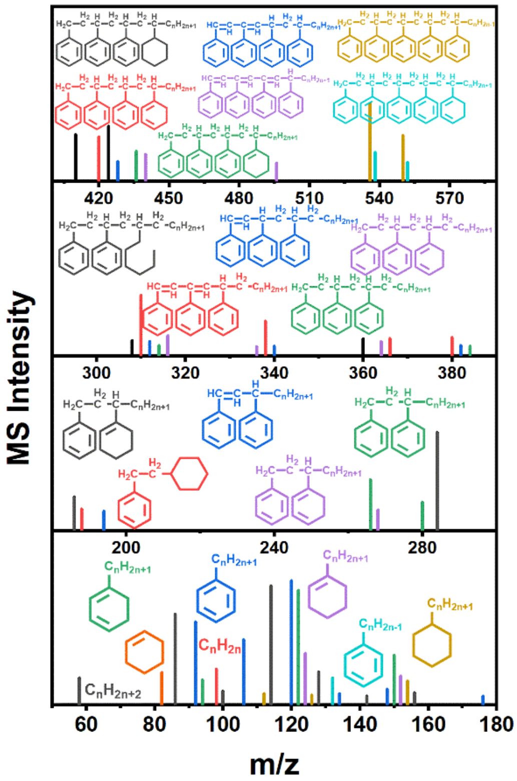

Supplementary Fig. 34. ESI-MS spectra compound identification for PCPS hydrogenolysis.

Reaction condition: v_{H2} = 100 ml/min, P_{H2} = 101 kPa, t = 12 min, P = 90 W.

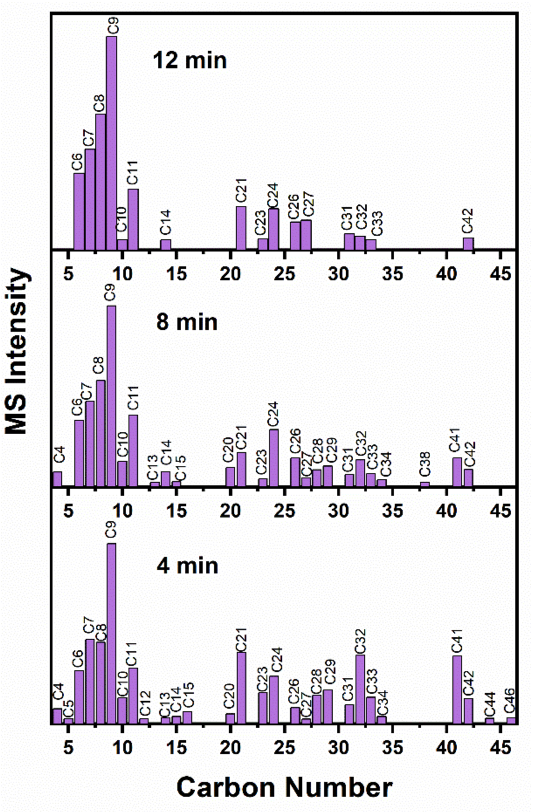

Supplementary Fig. 35. Carbon number distribution of PCPS liquid samples obtained from ESI-MS spectra.

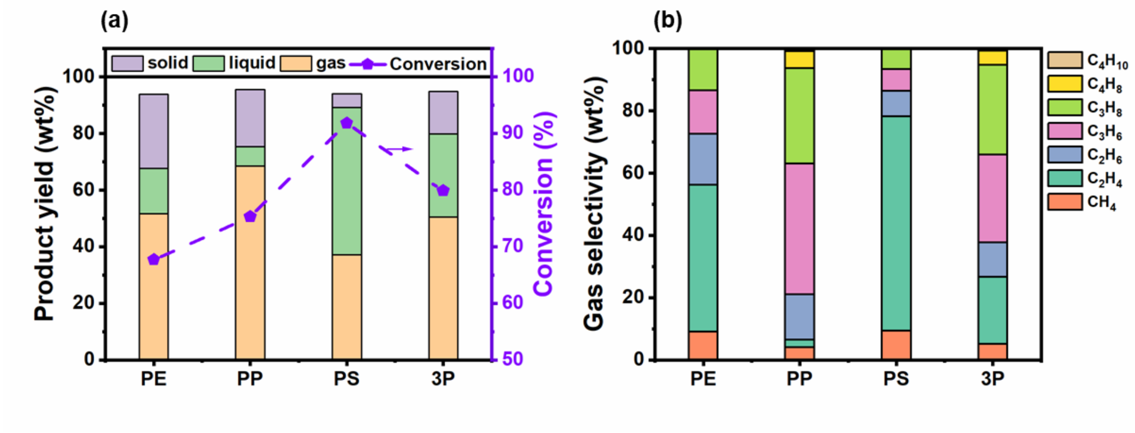

Supplementary Fig. 36. Reaction performance of PE, PP, PS and mixtures of the three substrates (3P). Reaction conditions: v_{H2} = 100 ml/min, P_{H2} = 101 kPa, t = 10 min, P = 90 W.

Supplementary Table 1. Demonstration of mass balance calculation. Reaction condition: ν_{H2} = 100 ml/min, P_{H2} = 101 kPa, t = 4 min and P = 90 W.

Terms	Weight (mg)
Quartz wool (m_{qw})	85.5
PS (m_{PS})	204.5
Reactor before (m_{rb})	40648.2
Reactor after (m_{ra})	40597.9
Reactor empty (m_{re})	40355.4
Uncollected solids (m_{us}=m_{rb}-m_{re}-m_{qw}-m_{PS})	2.8
$CHCl_3$ (m_{CF})	7.4
$CHCl_3$ extractant (m_{ex})	123.6
Filtered solids (m_{fs})	1.8
Solid residue (m_{sr})	110.5
m_{gas} (m_{rb}-m_{ra})	50.3
m_{liquid} (m_{ex}-m_{CF})	116.2
m_{solid} (m_{sr}-m_{qw}-m_{PS})	31
Uncounted (m_{uc}=m_{us}+m_{fs})	4.6
Error (m_{gas}+m_{liquid}+m_{solid}+m_{uc})/m_{PS}	1.17%

Supplementary Table 2. Summary of mass balance for PS hydrogenolysis reaction.

Entry	t (min)	v_{H2} (ml/min)	P_{H2} (kPa)	Power (W)	m_{PS} (mg)	m_{gas}+m_{liquid}+m_{solid}+m_{uc} (mg)	Error (%)
1	2	100	101.0	90	202.5	197.2	2.61
2	4	100	101.0	90	204.5	202.1	1.17
3	6	100	101.0	90	200.2	196.9	1.64
4	8	100	101.0	90	200.9	194.5	3.19
5	10	100	101.0	90	203.6	198.4	2.55
6	12	100	101.0	90	204.2	200.1	2.01
7	4	20	101.0	90	201.3	192.9	4.17
8	4	40	101.0	90	200.8	195.3	2.81
9	4	60	101.0	90	200.5	194.1	3.19
10	4	80	101.0	90	201.7	199.2	1.24
11	4	100	20.2	90	199.4	193.1	3.16
12	4	100	40.4	90	201.0	195.5	2.74
13	4	100	60.6	90	200.8	197.4	1.69
14	4	100	80.8	90	201.0	196.1	2.50
15	4	100	101.0	90	100.6	95.8	4.77
16	4	100	101.0	90	302.1	291.5	3.51
17	4	100	101.0	90	401.4	393.6	1.94
18	4	100	101.0	90	501.3	488.7	2.51
19	4	100	101.0	90	600.0	590.1	1.65

Supplementary Table 3. Band assignment for polystyrene IR spectra.

Wavenumber (cm^{-1})	Band assignment
3103	
3083	ν (CH) aromatic
3061	
3026	
2923	$-CH_2$ asymmetric stretching
2848	$-CH_2$ symmetric streching
1601	
1583	
1542	aromatic C-C stretching
1493	
1452	
1374	

Supplementary Table 4. Evaluation of v_{H2} on PS hydrogenolysis reaction performance.

Entry	v_{H2} (ml/min)	Conversion (%)	Mw (*10^3Da)		Yield (wt%)		
					Gas	Liquid	Solid
1	20	92.95	23.5	0.7	10.28	78.49	2.88
2	40	91.38	32.0	1.5	18.73	69.92	5.88
3	60	89.18	35.5	0.8	17.11	68.88	7.63
4	80	82.60	51.1	0.7	21.96	59.40	16.16
5	100	82.59	72.0	1.9	24.60	56.82	16.23

Supplementary Table 5. Evaluation of P_{H2} on PS hydrogenolysis reaction performance.

Entry	P_{H2} (kPa)	Conversion (%)	Mw (*10^3Da)		Yield (wt%)		
					Gas	Liquid	Solid
1	0	28.49	-	-	1.63	24.09	69.34
2	20.2	64.79	33.5	1.6	8.12	53.51	31.04
3	40.4	68.16	47.7	1.6	11.59	53.83	29.10
4	60.6	73.21	62.4	1.6	14.69	56.82	23.61
5	80.8	81.04	83.9	1.6	18.51	60.10	16.52
6	101	82.59	72.0	1.9	24.60	56.82	16.23

Supplementary Table 6. Evaluation of m_{PS} on PS hydrogenolysis reaction performance.

Entry	m_{PS} (mg)	Conversion (%)	Mw (*10^3Da)		Yield (wt%)		
					Gas	Liquid	Solid
1	100.6	95.23	41.3	0.2	58.85	36.38	0.00
2	204.5	82.59	72.0	1.9	24.60	56.82	16.23
3	302.1	80.40	65.8	1.5	21.19	47.34	17.84
4	401.4	79.97	66.6	1.6	18.63	59.39	18.09
5	501.3	76.76	75.8	1.5	12.63	61.62	20.73
6	600.0	74.70	85.5	1.5	11.48	61.57	23.65

Supplementary Table 7. Benson group increments for subgroups in polystyrene and formed products.

Group label	Identity	*ΔH*^{o} (kJ/mol)	*ΔS*^{o} (J/mol/K)	*ΔG*^{o} (kJ/mol)
m_1	C_{ar}-C	23.0	-32.1	32.6
m_2	C_{ar}-H	14.0	48.2	-0.4
m_3	C_{ar}-CH	-4.2	-50.9	11.0
m_4	$-CH_2-$	-21.0	39.4	-32.7
m_5	$-CH_3$	-42.7	127.2	-80.6
m_6	H_2	0	130.7	-38.9
m_7	CH_4	-74.9	188.7	-131.1
m_8	C_2H_2	226.7	200.9	166.8
m_9	C_2H_4	54.5	219.6	-10.9
m_{10}	C_2H_6	-84.0	229.6	-152.4
m_{11}	C_3H_6	20.4	266.9	-59.1
m_{12}	C_3H_8	-104.7	269.0	-184.9

Supplementary Table 8. Summary of thermodynamic property contributions for various reactions based on Benson group additivity.

Term	$\Delta H_f^0\ (\frac{kJ}{mol})$	$\Delta G^0 (\frac{kJ}{mol})$
m_{depoly}	-38.5	-52.7
m_{CH4}	-87.5	-108
m_{C2H4}	55.2	-3.4
m_{C2H6}	-62.3	-73.3
m_{C3H6}	47	-14.7
m_{C3H8}	-78.1	-101.6

Supplementary Table 9. Heat effect of H_2 plasma on the reactor temperature. Temperature measured with thermocouple directly after end of reaction.

Reaction time (min)	T (℃)
2	109
4	190
6	230
8	225
10	216
12	220

Supplementary Table 10. Additive thermodynamic properties (ΔH and ΔG) for PS hydrogenolysis reactions. The results were calculated by summing up all the gas formation contributions on a molar basis.

Reaction	$\Delta H_f^0\ (\frac{kJ}{mol})$	$\Delta G^0 (\frac{kJ}{mol})$
PS-2min	-57.5	-82.3
PS-4min	19.5	-28.7
PS-6min	25.2	-24.5
PS-8min	38.0	-15.7
PS-10min	24.9	-24.7
PS-12min	32.6	-19.0

Supplementary Table 11. Summary of selected PCPS hydrogenolysis performance.

Entry	t (min)	Conversion (%)	Molecular weight			Yield (wt%)		
			Mw (Da)	Mn (Da)	PDI	Gas	Liquid	Solid
1	4	64.69	98100	34200	2.87	7.42	53.66	28.87
2	8	82.78	62100	22000	2.82	28.73	50.61	11.85
3	12	85.25	19500	7640	2.55	40.99	38.99	7.33

Supplementary Table S12. Mass balance for PCPS hydrogenolysis.

Entry	t (min)	m_{PS} (mg)	$m_{gas}+m_{liquid}+m_{solid}+m_{uc}$ (mg)	Error (%)
1	2	213.0	205.3	3.60
2	4	203.3	196.3	3.44
3	6	199.3	188.8	5.27

Supplementary Table S13. Comparisons between NTP-assisted and conventional plastic hydrogenolysis methods.[3,18-23]

Substrates	Catalyst	Reactor type	T	Time	Pressure	Major products	Reference
PS	N/A	Continuous packed bed	RT	2-12 min	1 bar	C_2H_4	This work
HDPE	Pt/C, zeolite	Continuous packed bed	RT	2-12 min	1 bar	C_2H_4, C_2H_6	Ref 18
HDPE/LDPE	Pt/Al_2O_3	Batch autoclave	280 °C	3-24 h	N/A	Alkylaromatics	Ref 3
LDPE	Ru/C	Parr reactor	200-225 °C	16 h	20 bar	Liquid alkanes	Ref 19
PP+mixed PE	Ru/C	Parr reactor	200-250 °C	16 h	20-50 bar	Liquid alkanes	Ref 20
HDPE	Ru/C	Parr reactor	220 °C	1 h	60 bar	Fuels & lubricants	Ref 21
LDPE	Ru-WZr	Parr reactor	250 °C	2 h	30 bar	Diesel/wax	Ref 22
PP	Ru/TiO_2	Parr reactor	250 °C	3-40 h	50 bar	Lubricant	Ref 23

Supplementary references

1. Benson, S. W. *et al.* Additivity rules for the estimation of thermochemical properties. *Chem. Rev.* **69**, 279-324 (1969).

2. Cohen, N. Revised group additivity values for enthalpies of formation (at 298 K) of carbon–hydrogen and carbon–hydrogen–oxygen compounds. *J. Phys. Chem. Ref. Data.* **25**, 1411-1481 (1996).

3. Zhang, F. *et al.* Polyethylene upcycling to long-chain alkylaromatics by tandem hydrogenolysis/aromatization. *Science* **370**, 437-441 (2020).

4. Rodrigues, F., Pascoa, J. & Trancossi, M. Heat generation mechanisms of DBD plasma actuators. *Exp. Therm. Fluid Sci.* **90**, 55-65 (2018).

5. El-Shafie, M., Kambara, S. & Hayakawa, Y. Study of the reactor temperature effect on H_2 production from steam decomposition using DBD plasma. *Energy Rep.* **6**, 45-51 (2020).

6. Wang, J. *et al.* One-step plasma-enabled catalytic carbon dioxide hydrogenation to higher hydrocarbons: significance of catalyst-bed configuration. *Green Chem.* **23**, 1642-1647 (2021).

7. Constantinou, L. & Gani, R. New group contribution method for estimating properties of pure compounds. *AIChE J.* **40**, 1697-1710 (1994).

8. Moiseeva, N. F., Dorofeeva, O. V. & Jorish, V. S. Development of benson group additivity method for estimation of ideal gas thermodynamic properties of polycyclic aromatic hydrocarbons. *Thermochim. Acta* **153**, 77-85 (1989).

9. Wang, L., Yi, Y., Guo, H. & Tu, X. Atmospheric pressure and room temperature synthesis of methanol through plasma-catalytic hydrogenation of CO_2. *ACS Catal.* **8**, 90-100 (2018).

10. Winter, L. R. & Chen, J. G. N_2 fixation by plasma-activated processes. *Joule* (2020).

11. Wu, Z. *et al.* Enhanced energy efficiency and reduced nanoparticle emission on plasma catalytic oxidation of toluene using Au/γ-Al_2O_3 nanocatalyst. *Chem. Eng. J.* **427**, 130983 (2022).

12. Zhu, T., Li, R., Ma, M. & Li, X. Influence of energy efficiency on VOCs decomposition in non-thermal plasma reactor. *Inter. J. Environ. Sci. Technol.* **14**, 1505-1512 (2017).

13. Ma, T., Jiang, H., Liu, J. & Zhong, F. Decomposition of benzene using a pulse-modulated DBD plasma. *Plasma Chem. Plasma Process.* **36**, 1533-1543 (2016).

14. Ma, Y., Wang, Y., Harding, J. & Tu, X. Plasma-enhanced N_2 fixation in a dielectric barrier discharge reactor: Effect of packing materials. *Plasma Sources Sci. Technol.* (2021).

15. Pei, X., Gidon, D. & Graves, D. B. Specific energy cost for nitrogen fixation as NOx using DC glow discharge in air. *J.Phys. D: Appl. Phys.* **53**, 044002 (2019).

16. Chen, G., Liu, S., Chen, S. & Qi, Z. FTIR spectra, thermal properties, and dispersibility of a polystyrene/montmorillonite nanocomposite. *Macromol. Chem. Phys.* **202**, 1189-1193 (2001).

17. Wu, H.-D., Wu, S.-C., Wu, I.-D. & Chang, F.-C. Novel determination of the crystallinity of syndiotactic polystyrene using FTIR spectrum. *Polymer* **42**, 4719-4725 (2001).

18. Yao, L., King, J., Wu, D., Chuang, S. S. & Peng, Z. Non-thermal plasma-assisted hydrogenolysis of polyethylene to light hydrocarbons. *Catal. Commun.* **150**, 106274 (2021).

19. Rorrer, J. E., Beckham, G. T. & Román-Leshkov, Y. Conversion of Polyolefin Waste to Liquid Alkanes with Ru-Based Catalysts under Mild Conditions. *JACS Au* (2020).

20. Rorrer, J. E., Troyano-Valls, C., Beckham, G. T. & Román-Leshkov, Y. Hydrogenolysis of Polypropylene and Mixed Polyolefin Plastic Waste over Ru/C to Produce Liquid Alkanes. *ACS Sustain. Chem. Eng.* **9**, 11661-11666 (2021).

21. Jia, C. *et al.* Deconstruction of high-density polyethylene into liquid hydrocarbon fuels and

lubricants by hydrogenolysis over Ru catalyst. *Chem Catal.* (2021).

22. Wang, C. *et al.* Polyethylene Hydrogenolysis at Mild Conditions over Ruthenium on Tungstated Zirconia. *JACS Au* (2021).

23. Kots, P. A. *et al.* Polypropylene Plastic Waste Conversion to Lubricants over Ru/TiO_2 Catalysts. *ACS Catal.* **11**, 8104-8115 (2021).
